# Model acetylcholinesterase‐Fc fusion glycoprotein biotechnology system for the manufacture of an organophosphorus toxicant bioscavenging countermeasure

**DOI:** 10.1002/btm2.10666

**Published:** 2024-04-25

**Authors:** Thomas G. Biel, Talia Faison, Alicia M. Matthews, Uriel Ortega‐Rodriguez, Vincent M. Falkowski, Edward Meek, Xin Bush, Matthew Flores, Sarah Johnson, Wells W. Wu, Mari Lehtimaki, Rong‐Fong Shen, Cyrus Agarabi, V. Ashutosh Rao, Janice E. Chambers, Tongzhong Ju

**Affiliations:** ^1^ Office of Biotechnology Products, Office of Pharmaceutical Quality, Center for Drug Evaluation and Research, Food and Drug Administration Silver Spring Maryland USA; ^2^ Department of Comparative Biomedical Sciences, Center for Environmental Health Sciences College of Veterinary Medicine, Mississippi State University Mississippi State Mississippi USA; ^3^ Department of Biomedical and Pharmaceutical Sciences College of Pharmacy, University of Rhode Island Kingston Rhode Island USA; ^4^ Facility for Biotechnology Resources Center for Biologics Evaluation and Research, United States Food and Drug Administration Silver Spring Maryland USA

**Keywords:** acetylcholinesterase fusion protein, biomanufacturing, bioscavenger, glycosylation, organophosphorus toxicant

## Abstract

Organophosphate (OP) toxicants remain an active threat to public health and to warfighters in the military. Current countermeasures require near immediate administration following OP exposure and are reported to have controversial efficacies. Acetylcholinesterase (AChE) fused to the human immunoglobulin 1 (IgG1) Fc domain (AChE‐Fc) is a potential bioscavenger for OP toxicants, but a reproducible AChE‐Fc biomanufacturing strategy remains elusive. This report is the first to establish a comprehensive laboratory‐scale bioprocessing strategy that can reproducibly produce AChE‐Fc and AChE(W86A)‐Fc which is a mutated AChE protein with reduced enzymatic activity. Characterization studies revealed that AChE‐Fc and AChE(W86A)‐Fc are *N*‐glycosylated dimeric fusion glycoproteins but only AChE‐Fc had the capability to bind to paraoxon (a model OP). This AChE‐Fc fusion glycoprotein bioprocessing strategy can be leveraged during industrial biomanufacturing development, while the research‐grade AChE‐Fc proteins can be used to determine the potential clinical relevance of the countermeasure against OP toxicants.

**Translational impact statement**

Current countermeasures against OP toxicants are reported to have controversial efficacy and are limited by requiring immediate administration following OP exposure. The development of new countermeasures against OPs is warranted. AChE fused to an IgG1 Fc domain (AChE‐Fc) is a potential countermeasure against OPs, but a reproducible AChE‐Fc biomanufacturing strategy remains elusive. This report describes the establishment of a comprehensive model biotechnology system for biomanufacturing AChE‐Fc. Using this tool, the applicability of the AChE‐Fc fusion protein as a potential clinical countermeasure against OP toxicity can be investigated for the public and for warfighters.

## 1 INTRODUCTION

Organophosphorus (OP) agents and compounds are a diverse group of chemicals that potently inhibit acetylcholinesterase (AChE), a serine hydrolase, leading to cholinergic crisis and death by respiratory failure.^1^ The only approved antidote in the United States for OP toxicity is an atropine and pralidoxime (2‐PAM) combination treatment that requires immediate administration following the onset of symptoms.^2^ Atropine is a competitive blocker for the muscarinic cholinergic receptor, while 2‐PAM serves as an OP‐inhibited AChE reactivator.^2^ Based on clinical and experimental data, the efficacy of 2‐PAM remains controversial because 2‐PAM treatment did not improve overall OP‐exposed patient outcomes and survival, and AChE inhibited by different OP agents subjected to 2‐PAM failed to reactivate.^3^, ^4^, ^5^ Pyridostigmine bromide (PB), a transient AChE inhibitor, is the only approved pretreatment for patients at risk of OP exposure that is presumed to prevent irreversible AChE inhibition by OP agents. However, pyridostigmine bromide requires administration every 8 h and the efficacy is dependent on the rapid use of the antidote following OP exposure.^6^ Alternative therapeutic and prophylactic strategies are in development including a truncated AChE protein fused to polyethylene‐glycol (AChE‐PEG) or an IgG1 Fc domain (AChE‐Fc), which are claimed to be potential stoichiometric bioscavengers for OP chemicals.^7^, ^8^, ^9^, ^10^, ^11^, ^12^ AChE‐PEG and AChE‐Fc have demonstrated OP bioscavenging properties with improved pharmacokinetic properties (clearance and elimination) as compared to a recombinant AChE protein in murine models.^11^, ^12^ However, the purity of the AChE‐PEG and AChE‐Fc proteins were not determined and the bioprocessing strategies were not fully described nor demonstrated to be reproducible. Furthermore, the physicochemical and biological properties, except for AChE activity, of the AChE fusion proteins were not investigated in those studies. These limitations do not support the capability to reproducibly manufacture AChE‐PEG and AChE‐Fc and accurately determine the applicability of the fusion proteins as potential clinical countermeasures against OPs.

During early‐stage drug development, structure–function relationships on candidate therapeutics are often assessed followed by protein engineering to improve function.^13^ For example, Fc‐engineering of monoclonal antibodies can reduce the risk for undesirable antibody effector functions.^14^ Though adverse reactions caused by AChE‐Fc administration has not been reported, acetylcholine deficiency caused by heightened AChE activity has been reported to have a potential physiological response that may lead to cognitive impairment as observed in Alzheimer disease models with acetylcholine deficiency.^15^, ^16^, ^17^ Here, this study has recognized this potential concern and proposed to manufacture a catalytically inactive AChE fusion protein referred to as AChE(W86A)‐Fc. The AChE(W86A)‐Fc fusion protein contains an AChE domain with a site‐specific tryptophan (W) to alanine (A) mutation at residue 86 (W86A) that reduces the acetylcholine hydrolytic activity of the enzyme, while retaining OP toxicant binding capabilities.^18^ Therefore, an AChE‐Fc protein with the W86A site specific mutation [AChE(W86A)‐Fc] may potentially have a lower risk for adverse reactions caused by elevated AChE activity as compared to AChE‐Fc protein. However, the capability to biomanufacture the AChE(W86A)‐Fc fusion protein and the potential OP bioscavenging properties of AChE(W86A)‐Fc remain unknown.

In this report, a model laboratory‐scale biotechnology system was established for biomanufacturing experimental Fc fusion proteins to investigate current gaps in manufacturing, biotechnology, and the control of drug substances and products. The AChE‐Fc protein was selected as proof‐of‐concept model protein for establishing the biotechnology system to facilitate the development of new clinically relevant therapeutic protein countermeasures against OPs. This evidence‐based AChE‐Fc bioprocessing strategy provides foundational biomanufacturing knowledge that can be leveraged to generate clinical‐grade AChE‐Fc material, which is needed to test the hypothesis that AChE‐Fc fusion proteins are clinically relevant bioscavengers for OP chemicals.^19^ Utilizing this AChE‐Fc model biotechnology system, a research‐grade AChE‐Fc fusion protein is available for the scientific community to investigate the potential clinical applicability of the AChE‐Fc fusion glycoproteins.

## 2 RESULTS

### 2.1 Establishment of the AChE‐Fc fusion protein biomanufacturing process

**FIGURE 1:**
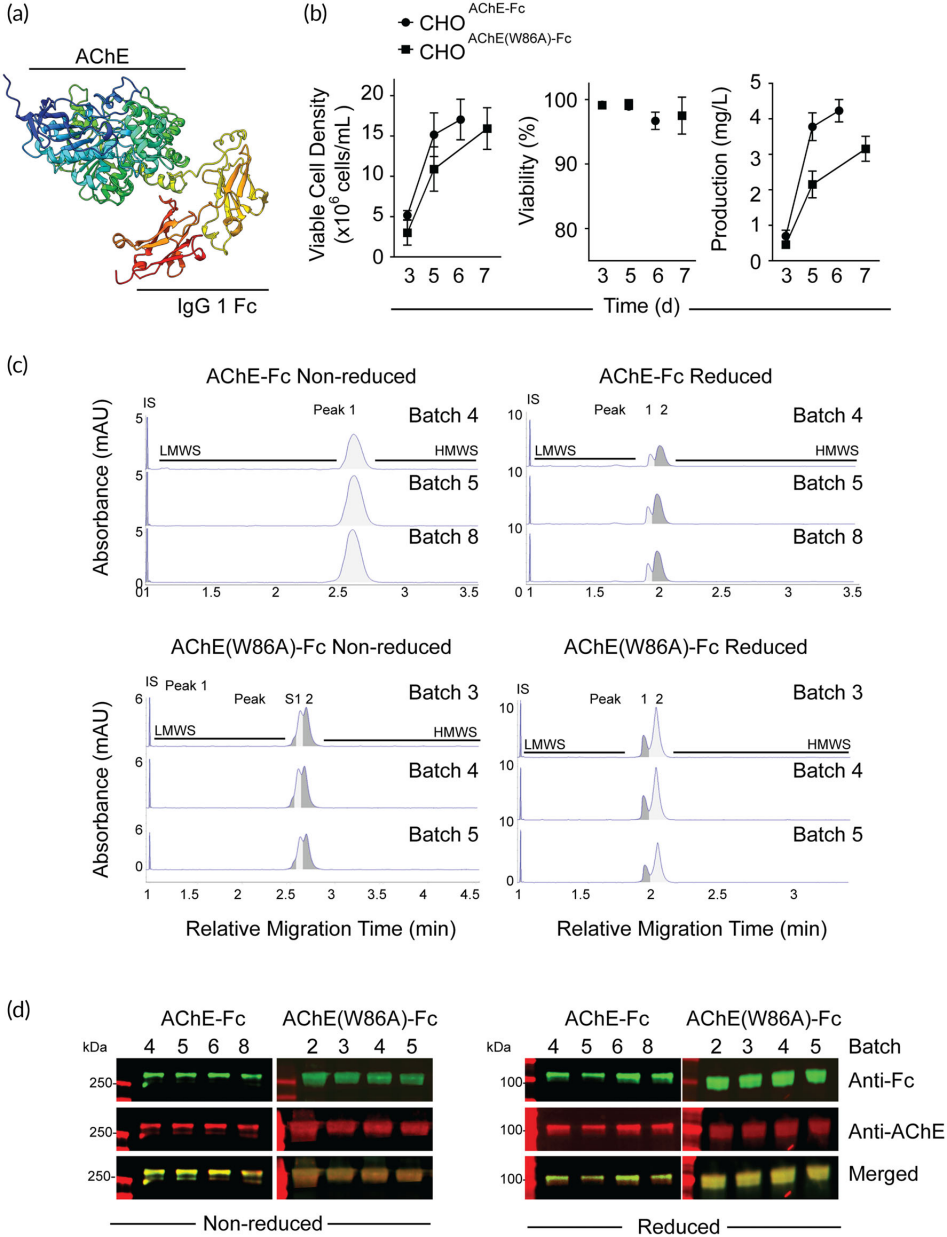
Biomanufacture, purity, and identity of AChE‐Fc and AChE(W86A)‐Fc. (a) Monomeric structural prediction of a truncated human acetylcholinesterase protein fused to a human IgG1‐Fc protein (AChE‐Fc). (b) The average viable cell density, cell viability, and fusion protein production of the CHO cells stably expressing AChE‐Fc (*n* = 4 batches) and AChE(W86A)‐Fc (*n* = 5 batches) monitored in triplicate per campaign. Dots represent the mean value with standard deviation error bars. (c) Representative electrophoretograms of AChE‐Fc and AChE(W86A) under nonreducing and reducing conditions. Delineations in the grayscale within the peaks indicates calculated area under curves for quantification. Each batch of AChE fusion protein was subjected to capillary SDS in triplicate. (d) Representative anti‐human IgG‐Fc (Green) and anti‐AChE (Red) immunoblots of AChE‐Fc and AChE(W86A)‐Fc following upstream and downstream processing to verify the presence of AChE and IgG‐Fc domains within the purified proteins.

The biomanufacture of therapeutic proteins commonly begins with the development of a cell substrate that synthesizes a protein of interest, and a bioprocessing strategy that can consistently manufacture the recombinant protein at the desired quantity and quality. To establish a model laboratory scale biomanufacturing system for investigating manufacturing and product quality‐related questions, the AChE‐Fc fusion protein was selected as a model fusion protein to generate evidence that will support the development and advancement of new clinical countermeasures against OP toxicity (Figure 1a).^20^ CHO cells are the most commonly used host cell line for the biomanufacture of therapeutic proteins.^21^ Therefore, CHO S cells that stably express AChE‐Fc (CHO^AChE‐Fc^) and AChE(W86A)‐Fc (CHO^AChE(W86A)‐Fc^) were generated and subjected to single cell cloning to further support the commercial relevance of this model biotechnology system.^22^ A single mammalian colony was selected and cultivated to generate the AChE‐Fc (CHO^AChE‐Fc^) and AChE(W86A)‐Fc (CHO^AChE(W86A)‐Fc^) master cell banks for biomanufacturing the AChE fusion proteins.

At least four bioprocessing campaigns per AChE fusion protein were monitored for variability in the cell viability, viable cell density, and productivity during the upstream process and the total protein recovery during the downstream process. The CHO^AChE‐Fc^ and CHO^AChE(W86A)‐Fc^ had a >94% cell viability throughout the duration of the campaigns with a maximum viable cell density greater than 10 million cells/mL on harvest day (Figure 1b and Table S1). Production of the AChE fusion proteins gradually increased throughout the campaigns until reaching 4.2 ± 0.1 mg/L for the CHO^AChE‐Fc^ and 3.2 ± 0.4 mg/L for the CHO^AChE(W86A)‐Fc^ (Figures 1b and S1a). The downstream process performed had two sequential chromatography steps that yielded ~36.5% AChE fusion protein recovery from the harvest media (Table S2).

Purity is a critical quality attribute for therapeutic proteins that is determined using a series of different analytical procedures that characterize different impurities due to their potential impact on drug product quality, safety, and efficacy.^23^ Product‐related impurities, such as protein aggregates and fragments, were investigated to confirm the model laboratory scale AChE fusion protein bioprocess could reproducibly manufacture AChE‐Fc and AChE(W86A)‐Fc at a purity of >90%. SDS‐PAGE, a low‐resolution method, revealed that the nondenatured purified proteins had a qualitative duplicate banding patterns at approximately 250 kDa under nonreducing conditions and 100 kDa under reducing conditions without the detection of any additional protein bands (Figure S1b). CE‐SDS was performed to quantify the presence of heavy and low molecular weight protein‐based impurities and confirmed the potential doublet banding patterns observed in the SDS‐PAGE gels (Figure 1c). AChE‐Fc was observed as a single broad peak with a 96.3% ± 3.0% purity under nonreducing conditions, and two partly overlapping peaks under reducing conditions that had a 96.2% ± 0.2% combined purity (Figure 1c and Table 1). AChE(W86A)‐Fc was observed under nonreducing conditions as a doublet peak with a modest shoulder that had a combined purity of 98.9% ± 0.4%, and two partly overlapping peaks under reducing conditions that had a 98.6% ± 0.2% combined purity. These data indicate the upstream and downstream process can biomanufacture these AChE fusion proteins at a purity greater than 95%, and the AChE fusion proteins are dimers that contained disulfide bond linkages.

**TABLE 1:** Quantification of AChE‐Fc and AChE(W86A)‐Fc electrophoretograms.

	AChE‐Fc (Batches = 4)	AChE(W86A)‐Fc (Batches = 3)				
	Peak 1 (P1)	Shoulder (S)	Peak 1 (P1)	Peak 2 (P2)	S + P1 + P2	
Nonreduced	96.3%	8.6%	39.3%	51.0%	98.9%	Ave.
	3.0%	0.7%	0.9%	0.5%	0.4%	SD
	3.2	8.3	2.2	1.0	0.4	CV (%)

	AChE‐Fc (Batches = 4)			AChE(W86A)‐Fc (Batches = 3)			
	Peak 1 (P1)	Peak 2 (P2)	P1 + P2	Peak 1 (P1)	Peak 2 (P2)	P1 + P2	
Reduced	70.7%	25.4%	96.2%	26.8%	71.9%	98.6%	Ave.
	0.4%	0.3%	0.2%	0.3%	0.3%	0.2%	SD
	3.6	11.7	2.9	1.0	0.5	0.25	CV (%)

*Note*: Values are derived from each fusion protein batch analyzed in triplicate.

Abbreviations: Ave., Average; CV, coefficient of variation; SD, standard deviation.

Identity is a critical quality attribute for therapeutic proteins that ensures drug product quality.^23^ Immunoblotting and tryptic peptide mapping were performed to confirm the identity of the AChE‐Fc and AChE(W86A)‐Fc proteins. Anti‐AChE and anti‐IgG Fc immunoblotting under reducing and nonreducing conditions confirmed the purified proteins were dimers that contained an AChE domain and an IgG Fc domain fused together, based on the overlapping fluorescent signatures at comparable sizes to the SDS‐PAGE results (Figures 1d and S1b,c). Under both nonreducing and reducing conditions, AChE(W86A)‐Fc proteins appeared as doublet bands or peaks, suggesting that AChE(W86A)‐Fc proteins may have higher heterogeneity than AChE‐Fc proteins. Peptide mapping confirmed the primary amino acid sequence with 83.58% and 85.82% sequence coverage to the theoretical primary amino acid sequence of AChE‐Fc and AChE(W86A)‐Fc, respectively (Figure S2). Moreover, peptide mapping verified the tryptophan to alanine residue mutation at amino acid in the AChE(W86A)‐Fc primary sequence as compared to the AChE‐Fc primary sequence (Figure S2). Collectively, these data indicated the establishment of a model laboratory‐scaled biotechnology system for the consistent biomanufacture of dimeric AChE‐Fc and AChE(W86A)‐Fc fusion proteins with a protein‐based purity greater than 94%.

### 2.2 *N*‐glycosylation characterization of AChE‐Fc fusion proteins

**FIGURE 2:**
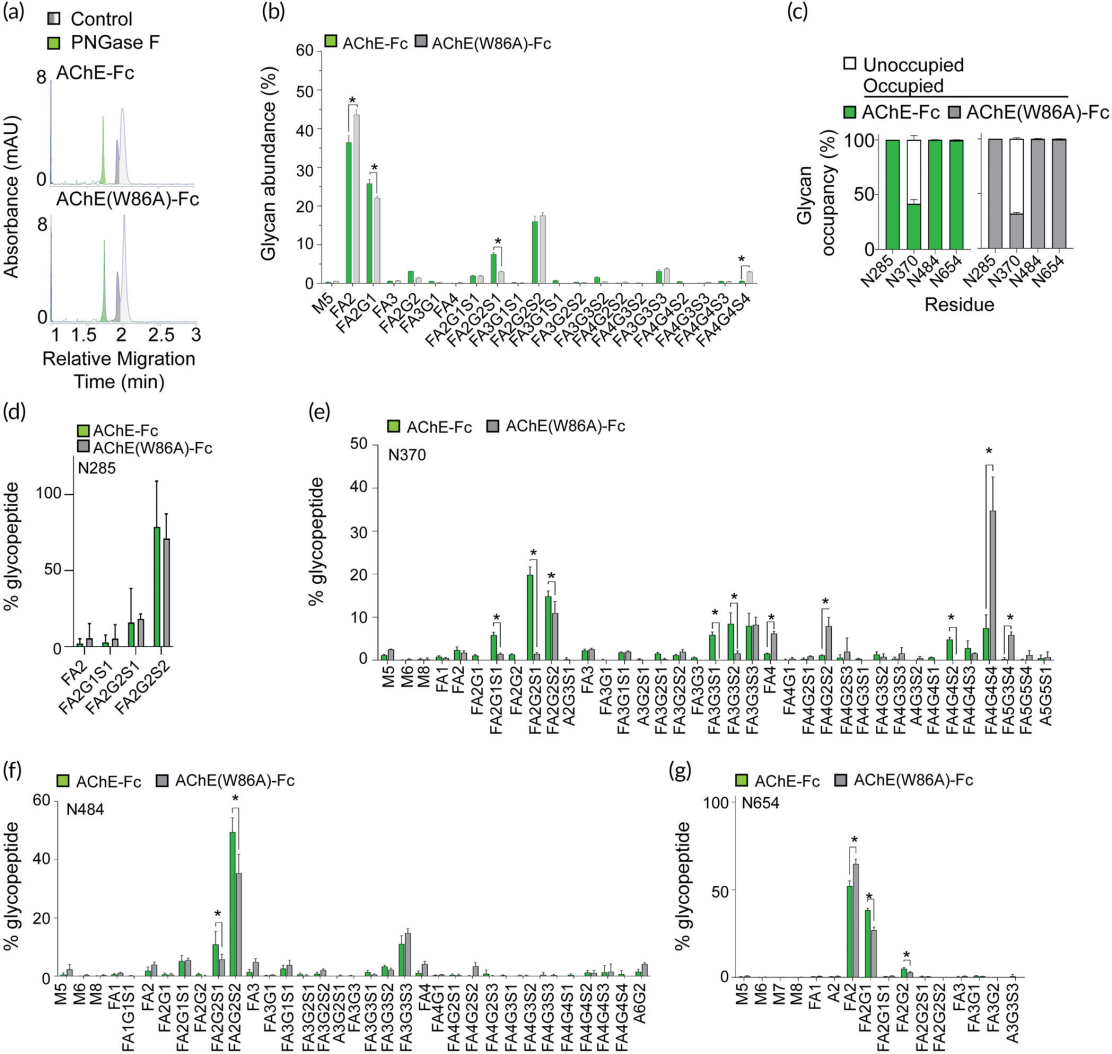
AChE‐Fc and AChE(W86A)‐Fc are *N*‐glycoproteins with micro‐ and macro‐ heterogeneity in *N*‐glycosylation. (a) Representative electropherograms of AChE‐Fc and AChE(W86A)‐Fc with and without PNGase F induced deglycosylation. (b) The average *N*‐glycosylation profile of AChE‐Fc and AChE(W86A)‐Fc from three different batches that were analyzed in triplicate. (c) *N*‐glycosylation occupancy of asparagine residues at positions 285, 370, 484 and 654 within the AChE‐Fc and AChE(W86A)‐Fc fusion proteins using pooled batches. (d–g) *N*‐glycan profile for each *N*‐glycosite using pooled AChE‐Fc fusion protein batches: (d) N265, (e) N370, (f) N484, and (g) N654. * indicates a *p* value of <0.05 using a two‐way ANOVA with *N*‐glycan and AChE‐Fc fusion protein as factors, and a Tukey's multiple comparison. Bars represent the mean value with standard deviation error bars.

*N*‐glycosylation is a common post‐translational modification on therapeutic proteins and is a critical quality attribute due to the impact on drug product safety and efficacy.^23^, ^24^ The primary amino acid sequences of the AChE‐Fc fusion proteins were searched for *N*‐glycosylation sites based on the Asn‐Xaa‐Ser/Thr (Xaa ≠ Pro) sequons.^25^ Each AChE‐Fc fusion protein had four predicted *N*‐glycan sites: N285 (Asn‐Asp‐Thr), N370 (Asn‐Glu‐Ser), N484 (Asn‐Tyr‐Thr), and N654 (Asn‐Ser‐Thr). To experimentally confirm the *N*‐glycosylation, the AChE fusion proteins were subjected to peptide‐*N*‐glycosidase F (PNGase F) digestion, which cleaves all *N*‐glycan species except for those that carry α1,3 core fucosylation found in invertebrates. Under reducing conditions, PNGase F induced de‐glycosylation caused the protein bands to migrate from ~100 to 75 kDa indicating the presence of *N*‐glycans on the AChE fusion proteins (Figure S3a). Moreover, the reduced AChE‐Fc fusion proteins observed to be a dual peak that migrated at ~2 min during CE‐SDS were transformed to a single peak with a faster migration time after PNGase F treatment (Figure 2a). These data support AChE‐Fc fusion proteins are heterogeneously glycosylated, and the difference between the apparent and theoretical molecular weight is mainly due to *N*‐glycosylation.

To establish the *N*‐glycosylation profiles of the AChE‐Fc fusion proteins, MALDI‐TOF/TOF‐MS was performed to structurally and quantitatively analyze the *N*‐glycan structures released from the AChE‐Fc fusion proteins. Twenty‐one different *N*‐glycan species were identified on both proteins and the relative abundance of FA2, FA2G1, FAG2S1, and FA4G4S4 were determined to be significantly different between manufactured AChE‐Fc fusion proteins, which is potentially due to the limited number of analyzed batches (Figures 2b and S3b,c). The major *N*‐glycan species identified were FA2 [AChE‐Fc 36.4%; AChE(W86A)‐Fc 43.6%], FA2G1 [AChE‐Fc 25.8%; AChE(W86A)‐Fc 22.0%], FA2G2S2 [AChE‐Fc 16.0%; AChE(W86A)‐Fc 17.5%], FA2G2S1 [AChE‐Fc 7.5%; AChE(W86A)‐Fc 3.0%], and FA3G3S3 [AChE‐Fc 3.1%; AChE(W86A)‐Fc 3.7%] (Tables S3 and S4). The *N*‐glycans with core fucosylation accounted for >99.0% (20/21) of the *N*‐glycan species with the M5 species being the only *N*‐glycan without core fucosylation. A variety of sialylated tri‐antennary and tetra‐antennary complex *N*‐glycans were identified and a collective relative abundance was greater than 9.0% for each AChE fusion protein. These data confirm that these AChE fusion proteins are heterogeneously *N*‐glycosylated.

Based on the primary sequences of AChE fusion proteins, each monomer may contain up to four *N*‐glycosylation sites.^25^, ^26^, ^27^ A glycoproteomic analysis was performed to determine *N*‐glycan occupancy and structures at each specific *N*‐glycosite in the AChE‐Fc fusion proteins. *N*‐glycans were identified on three asparagine residues in the AChE domain (N285, N370, and N484) and one asparagine residue (N654) in the IgG1 Fc domain (Supplementary Figure 2). *N*‐glycan occupancy at N285, N484, and N654 residues was greater than 99%, but only site N370 had variable *N*‐glycan occupancy at 41.6% ± 3.8% in AChE‐Fc and 31.8% ± 0.9% in AChE(W86A)‐Fc, which is consistent with the observed doublet banding patterns of SDS‐PAGE and the partly overlapping peaks of ce‐SDS (Figure S1b,c). Furthermore, different *N*‐glycan species were observed at each *N*‐glycosite (Figure 2d–g). Four different *N*‐glycan species were observed on the N285 residue of the AChE fusion proteins based on a series of complex biantennary species, with FA2GS2 being the most predominant, while the Fc *N*‐glycan site (N654) was occupied with 16 different *N*‐glycans, mostly neutral species such as FA2 followed by FA2G1 being the most predominant (Figure 2d,g). Interestingly, the N484 and N370 *N*‐glycosites of AChE fusion proteins, were identified to have over 30 different complex *N*‐glycan species including a variety of di‐, tri‐ and tetra‐antennary species. In both AChE‐Fc fusion proteins, FA2G2S2 was the most predominant at the N484 site, while the most predominant *N*‐glycans were FA2G2S1 for AChE‐Fc and FA4G4S4 for AChE(W86A)‐Fc at the N370 site (Figure 2e,f). With the limited batch analyses for each AChE‐Fc fusion protein, these data confirm micro‐ and macro‐heterogeneity of *N*‐glycosylation in AChE‐Fc and AChE(W86A)‐Fc fusion proteins.

### 2.3 Thermal stress‐induced AChE‐Fc fusion glycoprotein instability

**FIGURE 3:**
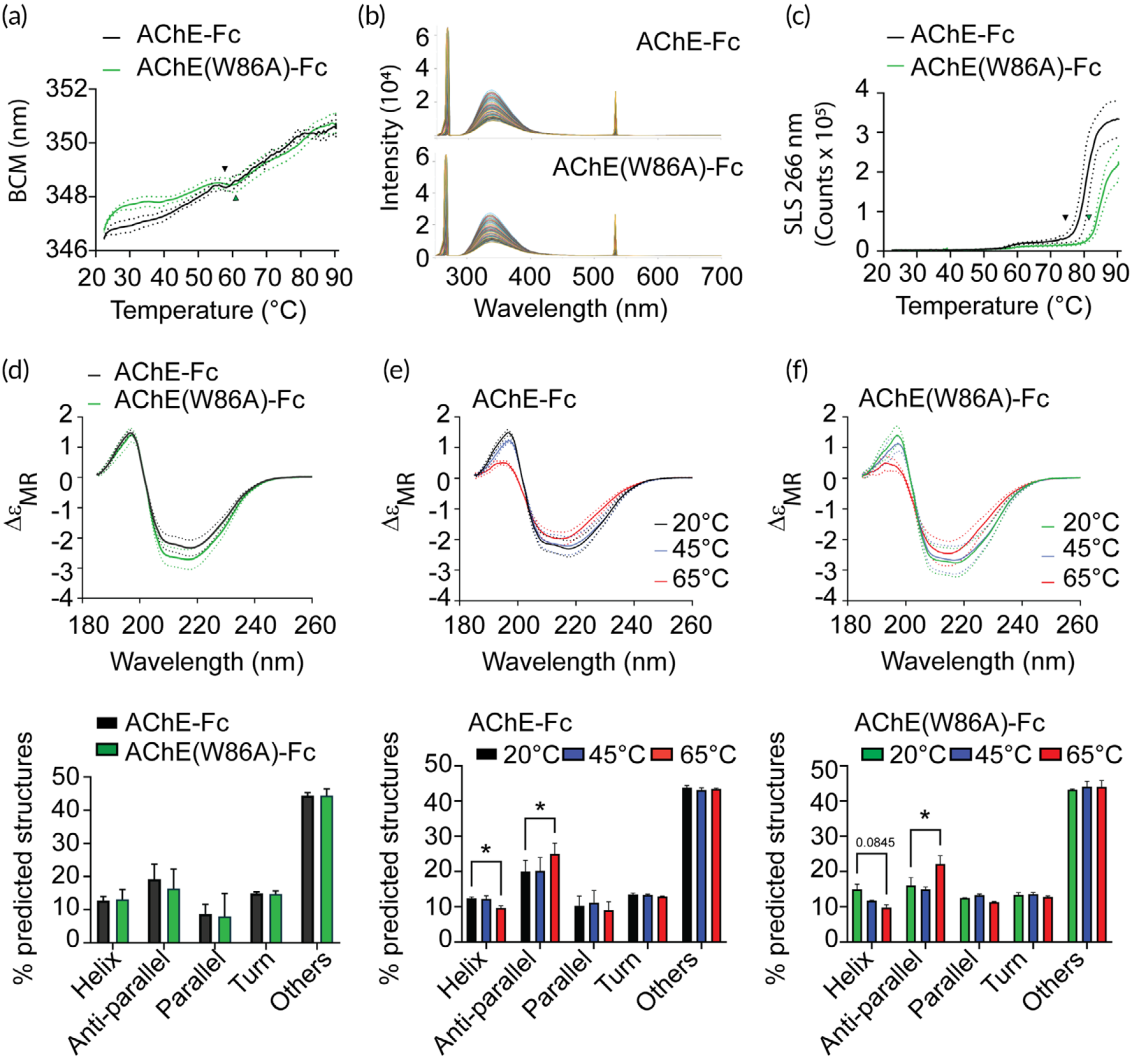
AChE‐Fc and AChE(W86A)‐Fc protein stability is sensitive to thermal stress. (a) Average barycentric mean (nm) showing the folding status of the AChE fusion proteins. Three batches were analyzed in duplicate, and the arrowheads indicate average protein melting temperature (*T*_m_). (b) Representative spectral changes of the AChE‐Fc fusion proteins during thermal ramping. (c) Average particle count using static light scattering at 266 nm during thermal ramping. Three batches were analyzed in duplicate, and the arrowheads indicate the average aggregation temperature at 266 nm (*T*_agg_ = 266 nm). (d) Average CD spectra of AChE fusion proteins (top) with predicted secondary structures (bottom). Three batches were analyzed in triplicate.(e, f) Average CD spectra of (e) AChE‐Fc and (f) AChE(W86A)‐Fc (top) with predicted secondary structure (bottom) at 20, 45, and 65°C. One batch was analyzed in triplicate. * indicates a *p* value of <0.05 using a two‐way ANOVA with secondary structure and temperature as factors, and a Tukey's multiple comparison. Solid lines represent the mean and dotted lines indicate the standard deviation. Bars represent the mean value and brackets report the standard deviations.

Protein thermal instability is one factor that determines the storage and shipment conditions of therapeutic proteins.^28^ UNcle is a high throughput, multifunctional protein stability screening system with the capability to monitor changes in the intrinsic fluorescence properties of proteins under thermal ramping conditions and static light scattering properties at 266 nm under thermal stress conditions.^29^, ^30^, ^31^, ^32^, ^33^, ^34^ Similar to previous reports, the barycentric mean (BCM) was identified from the intrinsic fluorescence profiles of the AChE‐Fc fusion proteins subjected to thermal ramping conditions to calculate the protein melting temperature (*T*_m_) and the onset of protein melting (*T*_m_ onset) (Figure 3a).^30^, ^32^ The calculated *T*_m_ was 55.1 ± 4.2°C for AChE‐Fc and 61.6 ± 6.4°C for AChE(W86A)‐Fc, while the calculated *T*_m_ onset was 45.8 ± 4.3°C for AChE‐Fc and 51.6 ± 9.9°C for AChE(W86A)‐Fc. Static light scattering of proteins at 266 nm has been claimed to identify the aggregation temperature (*T*_agg_) based on the transition of the *T*_agg_ 266 nm value during thermal ramping.^33^, ^34^ As the BCM decreased during thermal ramping, a corresponding increase in the SLS counts at 266 nm were qualitatively observed at 65, 75, and 90°C (Figure S4). The static light scattering counts during thermal ramping were used to calculate the *T*_agg_ at 266 nm to be 75.8 ± 1.5°C for AChE‐Fc and 80.2 ± 0.23°C for AChE(W86A)‐Fc (Figures 3c and S4).

As an orthogonal measurement of temperature induced protein unfolding, far‐ultraviolet circular dichroism (CD) was performed to identify and quantify secondary structure changes before and after thermal stress. The predicted secondary structures percentages of AChE‐Fc and AChE(W86A)‐Fc were determined to be statistically comparable in the absence of thermal stress (Figure 3d). The percentage of secondary structures in the AChE fusion proteins were comparable between 20 and 45°C but subjecting the proteins to 65°C cause the percentage of α‐helixes to decrease and anti‐parallel β‐sheets to increase (Figure 3e,f). Collectively, these data confirm that AChE‐Fc and AChE(W86A)‐Fc are sensitive to thermal stress in the current liquid formulation.

### 2.4 Prolonged thermal stress induced AChE‐Fc fusion glycoprotein instability

**FIGURE 4:**
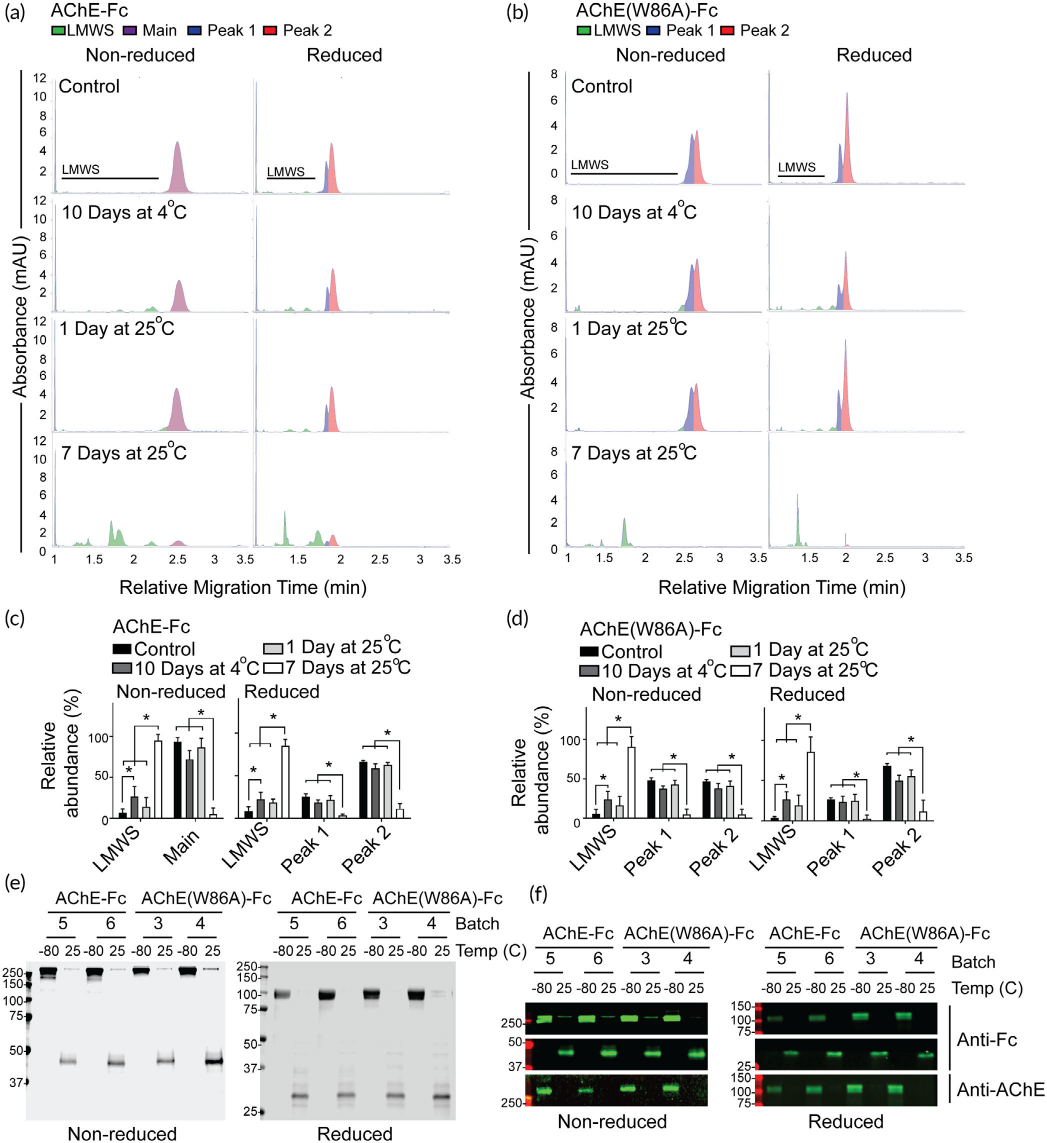
AChE‐Fc and AChE(W86A)‐Fc protein stability under prolonged thermal stress conditions. (a and b) Representative electropherograms of (a) AChE‐Fc and (b) AChE(W86A)‐Fc under nonreducing and reducing conditions exposed to different thermal stress conditions. Control is defined as storage at −80°C. (c and d) Area under the curve quantitation of (c) AChE‐Fc and (d) AChE(W86A)‐Fc electropherograms subjected to different thermal conditions. Three batches of each AChE fusion proteins were subjected to capillary SDS in triplicate (*n* = 9 per fusion protein per condition). * indicates *p* < 0.05 using a two‐way ANOVA with percentage and color‐delineated retention times as factors, and a Tukey's multiple comparison. Bars represent the mean value with standard deviation error bars. Low molecular weight species is abbreviated as LMWS. (e) Coomassie‐stained SDS‐PAGE gel of AChE‐Fc and AChE(W86A)‐Fc subjected to storage at −80°C and 7 days at 25°C. (f) Anti‐IgG Fc and anti‐AChE immunoblots of AChE‐Fc and AChE(W86A)‐Fc stored at −80°C and 7 days at 25°C.

Prolonged thermal stress studies were performed to quantify the formation of low and high molecular weight impurities for identifying an appropriate storage condition for the AChE fusion glycoproteins. As compared to a −80°C storage condition, AChE‐Fc and AChE(W86A)‐Fc stored for 10 days at 4°C and 7 days at 25°C had a significant increase in the formation of low molecule weight species (LWMS) (Figure 4a–d). No increasing trends or statistical differences in heavy molecular weight species were observed during these storage conditions at 4 and 25°C (Figure 4c,d). Under nonreducing CE‐SDS conditions, the average relative abundance of LMWS impurities of the AChE fusion proteins at −80°C storage was low [AChE‐Fc: 6.53% ± 5.0%; AChE(W86A)‐Fc: 5.6% ± 5.5%]. Storage of the AChE fusion proteins at 4°C for 10 days increased the LMWS impurities [AChE‐Fc: 23.9% ± 9.9%; AChE(W86A)‐Fc: 26.5% ± 11.8%], and at 25°C for 7 days substantially elevated the LMWS impurities [AChE‐Fc: 94.9% ± 7.5%; AChE(W86A)‐Fc: 90.7% ± 13.0%].

The AChE‐Fc fusion proteins subjected to prolonged thermal stress were analyzed to partly characterize the protein impurities using SDS‐PAGE and immunoblotting. Under nonreducing conditions, the AChE‐Fc fusion proteins subjected to 25°C for 7 days were observed by SDS‐PAGE to decrease in molecular weight from ~250 kDa to below 50 kDa, while the main protein bands under reduced conditions migrated from approximately 100 kDa to multiple bands lower than 37 kDa (Figure 4e). These data support that the thermal stress‐generated protein impurities were dimers that contain disulfide bonds. Anti‐IgG‐Fc and anti‐AChE immunoblotting revealed that some of the protein impurities contained a dimeric IgG‐Fc domain after storage at 25°C for 10 days, as indicated by the anti‐IgG‐Fc band decreasing from above 37 kDa under reducing conditions to below 37 kDa under nonreducing conditions (Figures 4f and S5). AChE‐Fc was not detectable above or below 37 kDa after 25°C for 7 days using immunoblotting (Figure S5). These data suggest that the AChE‐Fc fusion proteins in the current formulation should be stored at −80°C and subjecting the proteins to prolonged thermal exposure at 4 and 25°C will increase the quantity of LMWS impurities.

### 2.5 Acetylcholinesterase activity and OP binding capability of the AChE‐Fc fusion proteins

**FIGURE 5:**
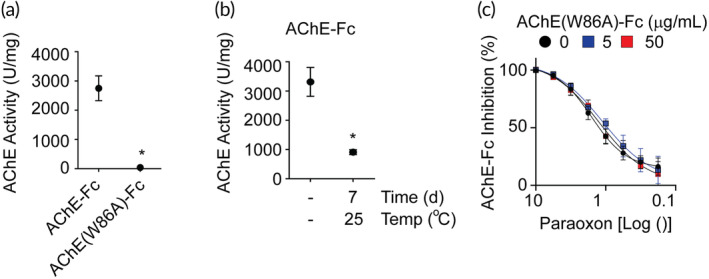
AChE‐Fc and AChE(W86A)‐Fc protein acetylcholinesterase hydrolytic capabilities and paraoxon binding properties. (a) The AChE hydrolytic capability of AChE‐Fc and AChE(W86A)‐Fc fusion proteins. Three batches of each fusion protein were analyzed in triplicate. (b) AChE hydrolytic capability of AChE‐Fc stored at −80°C and at 25°C for 7 days. Three batches of the AChE fusion protein were analyzed in triplicate (c) Paraoxon, an organophosphate (OP) toxicant, induced inhibition of AChE. AChE(W86A)‐Fc was preincubated with paraoxon at different concentrations prior to the addition to AChE‐Fc as an indirect approach to assess the capability of AChE(W86A)‐Fc to bioscavenge the toxicant. The paraoxon IC_50_ value for AChE‐Fc was 1.59 ± 0.14 μM. The IC_50_ values of AChE‐Fc using paraoxon incubated with 5 and 50 μg/mL of AChE(W86A)‐Fc was 1.18 ± 0.70 μM and 1.17 ± 0.22 μM, respectively. Pooled AChE‐Fc fusion protein batches were analyzed in triplicate. * indicates *p* < 0.05 using a Student's *t*‐test. Dots represent the mean with standard deviation error bars.

The biological activity of AChE‐Fc fusion proteins was investigated by characterizing the hydrolytic capability of the enzyme using a modified Ellman method. The AChE‐Fc and AChE(W86A)‐Fc fusion proteins were observed to have a 2751 ± 422.5 and 40 ± 3.64 U/mg hydrolytic capability, respectively (Figure 5a). Moreover, AChE‐Fc subjected to storage at 25°C for 7 days caused the hydrolytic capability to decrease from 3315.0 ± 493.4 to 912.4 ± 84.2 U/mg (Figure 5b). These data support that the site‐specific tryptophan to alanine mutation in the AChE domain of the AChE‐Fc fusion protein and thermal stress significantly reduce the acetylcholine hydrolytic capabilities.

Sarin, a potent OP toxicant, binding to AChE‐Fc based on an enzymatic inhibition assay has a reported *K*_*i*_ of 10.3 × 10^5^ M^−1^ min^−1^, which was comparable to the recombinant AChE protein.^12^ Diisopropylfluorophosphate, another OP toxicant, has been reported to have a *K*_*i*_ of 2.6 × 10^−4^ M^−1^ min for recombinant AChE(W86A) as compared 14.0 × 10^−4^ M^−1^ min for recombinant AChE protein.^18^ To determine the OP binding property and acetylcholine hydrolytic capability of AChE(W86A)‐Fc, the OP binding potential was characterized using both AChE‐Fc fusion proteins in the presence of paraoxon, a potent OP toxicant. The IC_50_ of paraoxon to inhibit the hydrolytic potential of the AChE‐Fc protein was calculated to be 1.59 ± 0.14 μM (Figure 5c). To qualitatively determine the OP binding potential of the enzymatically inactivated AChE(W86A)‐Fc protein, an indirect AChE assay was performed that incubated paraoxon with 5 and 50 μg/mL of AChE(W86A)‐Fc prior to quantifying the hydrolytic capability of AChE‐Fc. Theoretically, paraoxon‐bound AChE(W86A)‐Fc could decrease the inhibition of AChE‐Fc by paraoxon to support that AChE(W86A)‐Fc has OP toxicant binding potential. However, the IC_50_ of paraoxon incubated with AChE(W86A)‐Fc to inhibit AChE‐Fc was not statistically different than the IC_50_ of paraoxon alone to inhibit AChE‐Fc (Figure 5c). The IC50 of paraoxon to inhibit AChE‐Fc with incubation with at 5 and 50 μg/mL was 1.18 ± 0.7 and 1.17 ± 0.22 μM, respectively. Comprehensively, these data support the AChE‐Fc fusion glycoprotein manufactured from the model biotechnology system is a potential bioscavenger for OP toxicants.

## 3 DISCUSSION

AChE fused to an IgG1 Fc domain (AChE‐Fc) was reported to be a stoichiometric countermeasure against OP toxicants.^12^ This is the first report that describes the establishment of a comprehensive AChE‐Fc bioprocessing strategy for AChE‐Fc fusion glycoprotein production. This proof‐of‐concept model AChE fusion protein biotechnology system provides a framework for the biomanufacture of clinic‐grade material, which is required to investigate the applicability of the AChE‐Fc fusion protein as a countermeasure against OP toxicants for the public and for warfighters. This bioprocessing strategy can be leveraged to expedite the development of a costly industrial biomanufacturing process to acquire a clinical‐grade AChE‐Fc fusion protein. Our goal is to leverage this model to address current gaps in investigative strategies for chemistry, biomanufacturing, and control. Additional variations of this AChE fusion protein biotechnology system are in development that will identify methods and approaches for improving the biomanufacturing of safe and effective therapeutic protein drug products. These research‐grade AChE fusion proteins are available to the community to facilitate the development of new countermeasures against OPs.

This model AChE fusion protein biotechnology system is the first to demonstrate reproducible production of the AChE fusion proteins, but it possesses limitations that require further development and additional studies. The cell substrates (CHO^AChE‐Fc^ and CHO^AChE(W86A)‐Fc^) generated for this model system were identified to be low AChE fusion protein production cell lines with titers that range between 3 to 4 mg/L as compared to industrial cell substrates that biomanufacture proteins of interest at up to 10 g/L.^35^ To improve the productivity, new cell substrates need to be generated from a high producing AChE fusion protein cell line.^35^ The bioprocessing strategy requires optimization to improve productivity during the upstream processing and total protein recovery during downstream processing.^36^ Moreover, additional AChE‐Fc fusion protein characterization studies are warranted that quantify the levels of host cell proteins and DNA, and endotoxin impurities, which are additional critical quality attributes used to assess the overall purity of clinical therapeutic proteins. These biomanufacturing limitations are currently under investigation for the second‐generation AChE fusion protein biotechnology system.

Glycosylation is a common post translational modification on therapeutic proteins that is a common critical quality attribute for therapeutic proteins because of the impact *N*‐glycans can have on the stability, safety, and efficacy of drugs.^24^ This is the first report that characterized the *N*‐glycosylation status of the AChE fusion glycoproteins. The *N*‐glycan profile comparability between the AChE fusion proteins from at least three different batches supports the reproducibility of this model AChE‐Fc biotechnology system, but changes in the *N*‐glycosylation profile may occur during the further development of the biomanufacturing process, which requires optimization.^24^ Moreover, *N*‐glycoproteomic analysis confirmed the *N*‐glycan sites in addition to identifying the major *N*‐glycan structures and occupancy at each glycosite. N285, N484, and N654 *N*‐glycan sites were fully occupied with *N*‐glycan structural micro‐heterogeneity while one N370 glycosite was only partially occupied. As for the observed differences in structures of *N*‐glycans between AChE‐Fc and AChE(W86A)‐Fc fusion proteins, we speculate it is cell substrate‐specific or within the variability of the analytical method as opposite to their possible different structures caused by the W86A mutation. As this report is the first to characterize the *N*‐glycosylation status of the AChE‐Fc fusion glycoproteins, the impact that the *N*‐glycans have the on the stability, safety, potency, and efficacy of the AChE fusion proteins remains unclear and warrants additional investigations.

Therapeutic proteins that are sensitive to thermal conditions can be maintained by cold chain storage and delivery, which requires appropriate infrastructure that is expensive to build and maintain.^37^ We observed that AChE fusion proteins required cold chain storage at −80°C in the current formulation, which is a significant limitation due to storage and shipment challenges for the public and for warfighters. We speculate that the formation of LMWS during storage for 10 days at 4°C and 7 days at 25°C is potentially caused by using a nonoptimal formulation or the presence of trace host cell protein impurities in the purified protein. Formulation development studies have been reported to improve the stability of therapeutic proteins during transport and storage under different conditions and downstream optimization has been reported to remove trace amounts of a host cell protein that cleaved a recombinant protein under prolonged storage conditions.^38^, ^39^ Formulation development and downstream optimization studies are needed to improve the stability of the AChE fusion proteins, which is common for protein therapeutics. Studies are ongoing to identify the root cause leading to protein instability of the AChE fusion proteins, which may generate evidence that improves the quality of a AChE fusion protein during transport and storage.

This is the first report to generate evidence that supports the establishment of a comprehensive AChE‐Fc fusion glycoprotein biotechnology model. Our findings demonstrate that a two‐step downstream process was analytically comparable in protein recovery and protein‐based purity for biomanufacturing two different AChE‐Fc glycoproteins to support further refinement of the process. Additionally, the purified AChE‐Fc and AChE(W86A)‐Fc fusion proteins from the limited number of manufactured batches were deemed to be analytically comparable in secondary structure, thermal stress induced denaturation, and *N*‐glycosylation. The W86A point mutation in the AChE domain of the fusion glycoprotein was determined to suppress both hydrolysis of acetylcholine and paraoxon binding, which has been previously reported.^11^, ^18^, ^40^, ^41^ Alternative protein engineering strategies for the AChE‐Fc fusion protein are warranted to address potential adverse reactions that may be caused by heightened AChE activity and acetylcholine depletion follow AChE‐Fc administration. Protein modeling studies are on‐going to identify candidate AChE‐Fc fusion glycoprotein variants with improved thermal stability, reduced AChE hydrolytic activity, and enhanced OP binding capabilities for subsequent model AChE‐Fc fusion glycoprotein biotechnology systems.

## 4 CONCLUSION

This model AChE fusion protein biotechnology system is the first bioprocessing strategy that can reproducibly generate a purified AChE‐IgG1 Fc fusion glycoprotein. This feasible bioprocessing strategy supports the capability for the rapid development and biomanufacture of AChE fusion glycoproteins for public emergencies and warfighter preparedness. The AChE‐Fc fusion proteins manufactured from this system were subjected to analytical methods that characterized the physicochemical properties and biological activity of the molecules to support further development and testing of the potential countermeasure against OPs toxicants. Using this research‐grade AChE‐Fc protein, the potential of the countermeasure to be a therapeutic or prophylactic can be determined, and bioassays that control the quality of the potential product can be developed. Collectively, this report demonstrates the capability to biomanufacture AChE‐Fc for the development of reagents, therapeutics, and prophylactics for the advancement of clinically relevant countermeasures against OP toxicants.

## 5 MATERIALS AND METHODS

Detailed descriptions of each method are provided in the Supplementary Material section.

### 5.1 Protein modeling

The AChE fusion protein model was generated using algorithms and the quality of the model was compared to the highest quality model from Alphafold.^42^ Glycans were added to their respective sites using Glycam (Complex Carbohydrate Research Center, University of Georgia) and the glycoprotein was energy minimized using Amber12.^43^ Monomer subunits were docked using the Galaxy (Seoul National Lab) server and glycosylated subunits were aligned to the corresponding dimer template.

### 5.2 Cell line development and biomanufacturing

Stable Chinese hamster ovary (CHO) cells expressing the AChE fusion proteins were monoclonal derived and banked as previously described.^44^ One vial from each cell bank was expanded to inoculate the multiple 1 L shake flasks that were pooled during harvest during fed batch campaign. The campaign duration was 6 or 7 days based on the cell viability remaining >85%.

### 5.3 Purity and identity

Purity was qualitatively monitored for the presence of a single or duplicate protein banding pattern without any additional protein bands using SDS‐PAGE and Coomassie blue staining. Protein‐based purity was quantified by capillary electrophoresis SDS (ce‐SDS). Anti‐AChE and anti‐IgG Fc immunoblotting was performed to support the purified protein contained both the AChE and IgG1 Fc domains, while peptide mapping quantified the sequence coverage of the AChE‐Fc and AChE(W86A)‐Fc primary amino acid sequences to the theoretical amino acid sequences.

### 5.4 *N*‐glycosylation site prediction

To predict potential *N*‐glycosylation sites, the peptide sequences of AChE‐Fc fusion proteins were searched using NetNGlyc‐1.0 (Technical University of Denmark). Site prediction was performed based on the Asn‐Xaa‐Ser/Thr (Xaa ≠ Pro) sequons, and the occupation was predicted based on a threshold of 0.5 *N*‐glycosylation potential score.

### 5.5 *N*‐glycosylation profiling and *N*‐glycoproteomics

*N*‐glycans were isolated by a Filter Aided *N*‐Glycan Separation (FANGs) approach as described.^44^, ^45^ Briefly, PNGase F released *N*‐glycans were reduced, and permethylated as described, prior to analysis by matrix‐associated laser desorption/ionization‐time‐of‐flight—time‐of‐flight (MALDI‐TOF‐TOF) mass spectrometry on a Bruker UltrafleXtreme using 2,5‐dihydroxybenzoic acid matrix. *N*‐glycosylation profiles were obtained by calculating the glycan peak area and determining the relative precent abundance for each batch.

Tryptic *N*‐glycopeptides were prepared by a filter‐aided sample preparation approach (FASP) as described.^46^ Glycopeptides were analyzed using an Ultimate nanoLC coupled to a Thermo Fisher Fusion Orbitrap Liquid Chromatography Mass Spectrometer (LC–MS/MS) with oxonium‐triggered EThcD for glycopeptide fragmentation. Tandem mass spectra files were searched with proteome discoverer 2.4 using the protein sequences of AChE‐Fc, AChE(W86A)‐Fc, and the Uniprot Cricetulus griseus reference proteome. *N*‐glycoproteomic searches were performed using Protein Metrics Byonic/Byologic suite using the protein sequences of AChE‐Fc, and AChE(W86A)‐Fc, and a glycan database consisting of 182 human *N*‐glycans with no multiple fucose. Total peak areas from the extracted ion chromatograms (XICs) of each glycopeptide were converted to precent abundance by dividing the peak area of the respective *N*‐glycopeptide with the sum of peak area of all corresponding *N*‐glycopeptides and the native peptides at each site. Occupancy was determined by comparing the percent abundance of the *N*‐glycopeptides to the total corresponding peptide (*N*‐glycopeptide + native peptide).

### 5.6 Melting temperature and the secondary structure

Thermal ramping studies to assess the melting and aggregation temperatures of the fusion proteins were performed using the UNcle as described by the manufacturer (Unchained labs, Pleasanton, CA, USA). Far‐infrared ultraviolet CD was performed to obtain and quantify the secondary structures of the AChE‐Fc fusion proteins.

### 5.7 AChE activity

A commercial colorimetric assay (Cat # MAK119‐KT, Millipore Sigma) was used to measure the AChE activity of the fusion proteins. The activity was monitored using a standard Ellman assay with acetylthiocholine as substrate and 5,5′‐dithio(bis‐nitrobenzoic acid) (DTNB; Ellman's reagent) as the chromogen.^47^

### 5.8 OP inhibition assay

Each AChE fusion protein was diluted to yield about 1–1.2 Absorbance Units in 8 min in the Ellman method as previously described.^48^ The amount of protein to be used in each well was based on the protein levels. A vehicle to deliver the organophosphate, paraoxon, for the subsequent inhibition studies was determined by testing several combinations of aqueous and organic solvents. A final vehicle consisting of 1:1 (vol:vol), water:ethanol was selected. The absorbance was recorded at 50 s intervals for 8 min (for 10 readings) at 412 nm. Linearity of the reaction was determined to guarantee that substrate depletion did not occur. An indirect AChE assay was conducted to determine if the enzymatically inactivated AChE(W86A)‐Fc, could bind the OP (paraoxon). Diluted AChE(W86A)‐Fc fusion protein (5 and 50 μg/mL) was incubated (5–20 min) at 37°C with the same concentrations of paraoxon used to determine the IC_50_ value using AChE‐Fc. The active diluted fusion protein was added and incubated for 5 min as described above and AChE inhibition was determined. An observed reduction in AChE inhibition as compared to the AChE‐Fc protein alone would have suggested sequestration of the OP toxicant.

### 5.9 Reagents and statistical analyses

All other reagents were purchased from Sigma Aldrich. Statistical analyses were performed using GraphPad Prism 9 from at least two independently performed experiments. When two groups were compared, a Student's *t*‐test was performed. When >1 factor was analyzed, a two‐way ANOVA was performed with a Tukey's multiple comparison post‐hoc test to identify significant differences. A *p*‐value of <0.05 indicated statistical differences. The degree of freedom, *p*‐values, and test methods are provided in Table S5.

## AUTHOR CONTRIBUTIONS

**Thomas G. Biel:** Conceptualization; data curation; formal analysis; funding acquisition; investigation; methodology; project administration; resources; software; supervision; validation; visualization; writing – original draft; writing – review and editing. **Talia Faison:** Formal analysis; funding acquisition; investigation; methodology; project administration; software; validation; visualization; writing – review and editing. **Alicia M. Matthews:** Investigation; methodology; writing – review and editing. **Uriel Ortega‐Rodriguez:** Investigation; methodology; writing – review and editing. **Vincent M. Falkowski:** Investigation; methodology; writing – review and editing. **Edward Meek:** Investigation; methodology; writing – review and editing. **Xin Bush:** Investigation; methodology; writing – review and editing. **Matthew Flores:** Methodology; writing – review and editing. **Sarah Johnson:** Writing – review and editing. **Wells W. Wu:** Investigation; methodology; writing – review and editing. **Mari Lehtimaki:** Investigation; methodology; writing – review and editing. **Rong‐Fong Shen:** Investigation; methodology. **Cyrus Agarabi:** Conceptualization; funding acquisition; resources; writing – review and editing. **V. Ashutosh Rao:** Funding acquisition; resources; writing – review and editing. **Janice Chambers:** Formal analysis; funding acquisition; investigation; methodology; project administration; resources; validation; writing – review and editing. **Tongzhong Ju:** Conceptualization; funding acquisition; methodology; project administration; resources; supervision; visualization; writing – review and editing.

## FUNDING INFORMATION

The work described in this manuscript was supported by FDA/OCET's Medical Countermeasures Initiative (MCMi) grant OCET 2021‐1480 (US FDA), the CDER Advanced & Domestic Manufacturing Initiatives (US FDA), the Office of Pharmaceutical Quality's Centers of Excellence (US FDA), the Office of Biotechnology Products (US FDA), and an appointment to the Research Participation Program at the U.S. Food and Drug Administration administered by the Oak Ridge Institute for Science and Education through an interagency agreement between the U.S. Department of Energy and the U.S. Food and Drug Administration. The AChE assays were supported in part by the Center for Environmental Health Sciences, College of Veterinary Medicine, Mississippi State University.

## CONFLICT OF INTEREST STATEMENT

The authors have no competing interests to declare or conflicts of interest. The funding source had no part in the study design, data collection/analysis, decision to publish, or preparation of the manuscript. The views expressed in this article are those of the authors and do not necessarily reflect the official policy or position of the United States Food and Drug Administration and the Department of Health and Human Services, nor does mention of trade names, commercial products, or organizations imply endorsement by the United States Government.

### PEER REVIEW

The peer review history for this article is available at https://www.webofscience.com/api/gateway/wos/peer-review/10.1002/btm2.10666.

## Supporting information


**Table S1.** CHO Cell line characterization during the upstream production of AChE‐Fc and AChE(W86A)‐Fc.
**Table S2.** Efficiency of the two‐step downstream process for AChE‐Fc and AChE(W86A)‐Fc fusion protein purification.
**Table S3.** Relative percentages of *N*‐linked glycans identified using AChE‐Fc from at least 3 batches performed in triplicate.
**Table S4.** Relative percentages of *N*‐linked glycans identified using AChE(W86A)‐Fc from at least 3 batches performed in triplicate.
**Table S5.** Results, degrees of freedom, factors, and figures for statistical analyses.
**Figure S1.** The secreted AChE fusion proteins characterized for purity and identity.
**Figure S2.** Peptide map coverage of the AChE fusion proteins with *N*‐glycan sites, point mutation site, and sequence domains.
**Figure S3.** *N*‐glycan profiles of AChE‐Fc and AChE(W86A)‐Fc.
**Figure S4.** Static intrinsic fluorescent changes of AChE‐ Fc and AChE(W86A)‐Fc at 22, 65, 75, and 90°C.
**Figure S5.** Raw immunoblots of AChE fusion proteins subjected to storage for 7 days at 25°C.

## Data Availability

All the relevant data are within this publication and its Supplementary Material file.
