# Supplementary material for: Model acetylcholinesterase‐Fc fusion glycoprotein biotechnology system for the manufacture of an organophosphorus toxicant bioscavenging countermeasure
Source: Bioeng Transl Med. 2024 Apr 25;9(5):e10666. doi: 10.1002/btm2.10666 (PMC11561780; doi:10.1002/btm2.10666)
Supplement: Supplementary file 1 — Table S1. CHO Cell line characterization during the upstream production of AChE‐Fc and AChE(W86A)‐Fc. Table S2. Efficiency of the two‐step downstream process for AChE‐Fc and AChE(W86A)‐Fc fusion protein purification. Table S3. Relative percentages of N‐linked glycans identified using AChE‐Fc from at least 3 batches performed in triplicate. Table S4. Relative percentages of N‐linked glycans identified using AChE(W86A)‐Fc from at least 3 batches performed in triplicate. Table S5. Results, degrees of freedom, factors, and figures for statistical analyses. Figure S1. The secreted AChE fusion proteins characterized for purity and identity. Figure S2. Peptide map coverage of the AChE fusion proteins with N‐glycan sites, point mutation site, and sequence domains. Figure S3. N‐glycan profiles of AChE‐Fc and AChE(W86A)‐Fc. Figure S4. Static intrinsic fluorescent changes of AChE‐ Fc and AChE(W86A)‐Fc at 22, 65, 75, and 90°C. Figure S5. Raw immunoblots of AChE fusion proteins subjected to storage for 7 days at 25°C. [file BTM2-9-e10666-s001.docx]

**Model acetylcholinesterase-Fc fusion glycoprotein biotechnology system for the manufacture of an organophosphorus toxicant bioscavenging countermeasure**

**Supplementary Material**

**Methods and Materials**

*AChE-Fc fusion protein constructs and modeling*

The primary amino acid sequence for human Acetylcholinesterase (AChE) isoform H and the Fc region of IgG1-Fc were obtained from Uniprot (ID: P22303 and P01857-1). The primary amino acid sequence of AChE was modified by (i) replacing the collagen like structure from positions 545 to 583 with an ASEAP amino acid linker, (ii) exchanging the endogenous N-terminal secretory sequence with a Kappa signaling peptide, (iii) mutating the tryptophan residue at position 86 to alanine, and (iv) including a GGGSGGGS amino acid linker sequence followed by the IgG1 Fc region on the C-terminus. The primary amino acid sequence was then reverse translated and synthesized into a pcDNA3.1 vector backbone by Genscript (Piscataway, NJ) to generate an AChE(W86A)-Fc vector. To generate an AChE-Fc vector, the AChE-Fc nucleotide sequence without the point mutation at position 86 was synthesized by Genscript and cloned into the AChE(W86A)-Fc vector using standard molecular biology techniques.

The AChE-Fc and AChE(W86A)-Fc theoretical primary amino acid sequences are provided below and the **point mutation between the sequences was bolded and underlined.**

MVFTPQILGLMLFWISASRGEGREDAELLVTVRGGRLRGIRLKTPGGPVSAFLGIPFAEPPMGPRRFLPPEPKQPWSGVVDATTFQSVCYQYVDTLYPGFEGTEM**W/A**NPNRELSEDCLYLNVWTPYPRPTSPTPVLVWIYGGGFYSGASSLDVYDGRFLVQAERTVLVSMNYRVGAFGFLALPGSREAPGNVGLLDQRLALQWVQENVAAFGGDPTSVTLFGESAGAASVGMHLLSPPSRGLFHRAVLQSGAPNGPWATVGMGEARRRATQLAHLVGCPPGGTGGNDTELVACLRTRPAQVLVNHEWHVLPQESVFRFSFVPVVDGDFLSDTPEALINAGDFHGLQVLVGVVKDEGSYFLVYGAPGFSKDNESLISRAEFLAGVRVGVPQVSDLAAEAVVLHYTDWLHPEDPARLREALSDVVGDHNVVCPVAQLAGRLAAQGARVYAYVFEHRASTLSWPLWMGVPHGYEIEFIFGIPLDPSRNYTAEEKIFAQRLMRYWANFARTGDPNEPRDKAPQWPPYTAGAWWYVSLDLRPLEVRRGLRAQACAFWNRFLPKLLSATGGGGSGGGGSEPKSSDKTHTCPPCPAPELLGGPSVFLFPPKPKDTLMISRTPEVTCVVVDVSHEDPEVKFNWYVDGVEVHNAKTKPREEQYNSTYRVVSVLTVLHQDWLNGKEYKCKVSNKALPAPIEKTISKAKGQPREPQVYTLPPSTEEMTKNQVSLTCLVKGFYPSDIAVEWESNGQPENNYKTTPPVLDSDGSFFLYSKLTVDKSRWQQGNVFSCSVMHEALHNHYTQKSLSLSPGK

The FASTA sequence of the AChE-Fc fusion protein was 3D modeled using multiple modeling algorithms: AlphaFold2, SwissModel and Phyre2. Each model was individually compared across various quality metrics including QmeanisCO Global, Qmean Z, Cβ, All Atom IDDT, solvation and torsion scores. The highest quality model by all metrics was used to conduct protein-protein docking to generate hypothetical dimer structures. Multiple docking algorithms and servers were used to generate the most likely dimer structure, including HADDOCK, ZDOCK, CLUSPRO, and GALAXY. The top 5-10 models from each library were compared for biological likelihood and the best dimer was selected from GALAXY (Seoul National Lab), which used the AChE (PDB IDs: 4M0E and 6NTH) subunit as a template to generate the corresponding dimer structure. All PBD files were generated and optimized using PyMol. Dimer formation was also verified using AlphaFold Multimer to determine association similarity. Dimer structures were verified and compared using all quality metrics listed above, and the highest quality dimer was selected. With the dimer template generated, Glycam’s Glycoprotein builder (CCRC) was used to glycosylate a monomer for overlay with the dimer template. Each subunit was aligned to a corresponding subunit on the template and an energy minimization run with Amber12 was used to predict the mostly likely conformation of the glycosylated dimer.

*Cell substrate development*

FreeStyle CHO S cells were grown as described by the manufacturer (Cat# R80007, ThermoFisher). Cells were electroporated with the plasmid constructs and subjected to appropriate antibiotic selection. Enriched populations were seeded at 300 cells per well in a 6-well dish using semi solid ClonaCell-CHO CD Medium (Cat # 3815, StemCell Technologies). Cell colonies were then imaged, ranked by Fluorescent Total Sum Intensity, picked, and placed in 96-well plates containing FreeStyle CHO Medium (Cat # 12651014, ThermoFisher) using the ClonePix 2 Mammalian Colony Picker as described by the manufacturer (Molecular Devices). The cell population from each clone was counted on days 0 and 4 using a Celigo S cytometer (Nexcelom). On day 4, media from each well was sampled for protein titer via biolayer interferometry using Protein A biosensors and an Octet 96e as described by the manufacture (Sartorius). One colony from each line was selected for expansion, banking, and storage in liquid nitrogen.

*AChE-Fc fusion protein production*

Vials of CHO cell lines expressing the AChE fusion proteins were thawed and expanded to 1 L shake flasks in an environmentally controlled shake incubator under the following conditions: 5% CO2, 37°C, and 125 rpm. Flasks with FreeStyle CHO Medium (350 mL) were inoculated with 0.3 x 10^6^ cell/mL for a 6- or 7-day fed batch campaign. For fed batches, the cells were supplemented with bolus additions of 2X CD Efficient Feed C AGT, L-Glutamine, and NaOH on days 3 and 5. L-Glutamine (200 mM/L) was added to maintain a 6 mM/L concentration in the production media. Media pH was adjusted between 7 and 7.4 using 0.5 M NaOH additions. Feed C AGT was added to recover glucose between 4 and 6 mM/L while maintaining the Osmolality < 360 Osm/L. A BioProfile Flex analyzer (Nova Biomedical) was used for all cell culture media nutrient measurements. Batches were sampled on days 3, 5, and 6/7 to characterize cell viability via trypan blue staining and protein titer via Protein A biosensors (Cat# 18-5010, Sartorius, Germany) and an Octet 96e (Sartorius) as described by the manufacturer.

*AChE-Fc fusion protein purification*

Chromatography was performed using an Akta AVANT25 chromatography system with commercially available pre-packed columns (Cytiva, Marlborough, MA). Using a HiTrap Protein A HP column, AChE-Fc and AChE(W86A)-Fc were captured from the harvested media. The column was washed and equilibrated with 20 mM sodium phosphate at pH 7.4. The AChE fusion proteins were eluted from the Protein A column using a 1 M glycine at pH 3 and neutralized to pH 7.4 using a 1 M Tris solution at pH 9. Using a 30 kDa molecular weight cut-off filter, the protein A eluate was concentrated and subjected to buffer exchange using anion exchange binding buffer (10 mM Sodium Chloride and 25 mM Tris at pH 7.2). Anion exchange chromatography was performed using a Mono Q 5/50L column and a 30%-100% dynamic gradient with an elution buffer (350 mM Sodium Chloride and 25 mM Tris at pH 7.2). Recovered product was concentrated and subjected to a buffer exchange using formulation solution (1% sucrose, 100 mM sodium chloride, 25mM L-arginine hydrochloride, and 25mM sodium phosphate). A sample run was performed using an isocratic method with a buffer composed of 100 mM Sodium Sulfate, 100 mM Sodium Phosphate, 1 % IPA, pH 6.7 and at a flow rate of 0.35 mL/min. The protein was monitored using UV at 280 nm during purification, and the protein concentration in the eluant was determined using 280 nm absorption and a NanoDrop One Microvolume UV spectrophotometer (Cat# ND-ONE-W, Thermofisher).

*Purity by capillary electrophoresis SDS (CE-SDS)*

CE-SDS was performed using Maurice (Bio-techne, Minneapolis, MD) as described by the manufacturer. Briefly, purified protein (25 μg) treated with beta-mercaptoethanol (reduced) and iodoacetamide were diluted in Maurice CE-SDS PLUS, 1x Sample Buffer (Cat# 046-567, Bio-Techne) as described by the manufacturer. Samples were heated at 70 °C for 10 minutes before injection onto a Maurice CE-SDS PLUS Cartridge (Cat# PS-MC02-SP, Bio-techne) at 4.6 kV for 20 seconds and separated at 5.75 kV for 45 minutes (reduced) and 60 minutes (non-reduced).

*SDS-PAGE*

SDS-PAGE gels (Cat# 4569033, BIO RAD) were used to determine the purity and stability of the fusion proteins. Protein (1.5 μg) was loaded into each well and following electrophoresis the gel was stained with EZBlue Coomassie Stain (Cat#G1041-500ML, Sigma Aldrich) in accordance with manufacturer protocol. Gels were imaged using Azure 600 imaging system (Azure Biosystems, Dublin, CA).

*Immunoblotting*

Immunoblotting was performed using 15 microliters of cell media or 0.1 µg of purified protein. The membranes were probed using the following antibodies: anti-human Fc (Cat# NBP1-40876, Novus Biologicals), and anti-AChE (Cat# NB100-1519, Novus Biologicals). Membranes were imaged using Azure 600 imaging system (Azure Biosystems, Dublin, CA).

*N-glycosylation profiling*

N-glycans from the fusion glycoproteins (25 μg) were released using a Filter Aided N-glycan Separation (FANGS) approach with materials from an Abcam Filter Aided Sample Prep (FASP) protein digestion kit (Cat# ab270519, Abcam) as described [1]. Briefly, aliquots containing fusion glycoprotein were reduced with a urea-dithiothreitol (DTT) solution (100mM Tris/HCL pH 8.5, containing 10mM DTT) and the mixture was transferred to a 30 kDa molecular cutoff ultrafiltration device for centrifugation at 15,000 x g for 15 minutes. The filter bed was washed repeatedly using urea solution (8 M in 100mM Tris/HCL pH 8.5), and cysteine residues were alkylated with 40 mM iodoacetamide (IAA) for 20 minutes in the dark. The filter was washed repeatedly with the urea solution, and buffer-exchanged with 50 mM ammonium bicarbonate (pH 7.5). Finally, 50 mM ammonium bicarbonate containing 500U of glycerol-free PNGase F (Cat# P0709S, NEB) was added and the ultra-filtration cell was transferred to a fresh collection tube and incubated at 37°C for 21 hours. Released N-glycans were recovered by washing the filter LC-MS grade water, followed by centrifugation. Dry N-glycans purified with active charcoal micro spin column (Cat# 74-4800, Harvard Apparatus) and the N-glycan eluate was dried by vacuum centrifugation. N-glycans were reduced with a 10 mg/mL ammonia borane complex in water at 60°C for 1 hour. The samples were dried under vacuum, followed by a series of methanolic evaporations by adding 10% acetic acid in methanol and drying thoroughly under vacuum until excess reactants were removed. Reduced N-glycans were solubilized in 65 μL anhydrous dimethylsulfoxide, followed by 5 μL of LC-MS water, and 35 μL iodomethane and permethylated by solid-phase sodium hydroxide. Permethylated N-glycans were subsequently purified by C18 micro spin columns (Cat# 74-4601, Harvard Apparatus). The final eluate was dried by vacuum centrifugation and resuspended in 50% LC-MS methanol, mixed 1:1 with 2,5-dihydroxybenoic acid (10mg/mL containing 1mM NaCl in 30% ACN, 0.1% TFA) and spotted on a ground steel target. Glycans were analyzed using a Bruker UltrafleXtreme MALDI-TOF/TOF mass spectrometer. Mass measurements were obtained in positive ion reflector mode. Sodiated N-glycan peak intensity was normalized by converting to % abundance based on total glycan identifications.

*Peptide mapping and glycoproteomic analysis*

Three batches of Fc-fusion protein were pooled, and 50 µg of each protein were processed in triplicate with materials from an Abcam Filter Aided Sample Prep (FASP) protein digestion kit (Cat# ab270519, Abcam) as described [1]. Briefly, aliquots of glycoprotein were reduced in 200 µL of urea-DTT solution (100mM Tris/HCL pH 8.5, 10mM DTT) for 45 minutes at room temperature with end-over-end mixing. The reduced protein samples were transferred to a 30 kDa molecular cutoff ultrafiltration device and centrifuged at 15,000 x g for 15 minutes. Excess DTT was removed by repeatedly washing the filter unit with 200 µL urea solution (8 M in 100mM Tris/HCL pH 8.5) followed by centrifugation at 15,000 x g for 15 minutes. Cysteine residues were alkylated with 100 µL of iodoacetamide solution (40 mM iodoacetamide) followed by incubation at room temperature for 20 minutes in the dark. The filter unit was washed three times with 100 µL Urea solution, and then equilibrated with 50 mM ammonium bicarbonate (pH 7.5). Fc-fusion proteins were digested with 10 µg of Trypsin-ultra, Mass Spectrometry Grade (Cat# P8101S, New England Biolabs) in 100 µL of 50 mM ammonium bicarbonate at 37°C for 16 hours. Tryptic peptides were recovered by centrifugation at 15,000 x g for 15 minutes. The peptide filtrate was dried by vacuum centrifugation followed by reconstitution in 50 µL of water containing 0.1% Formic acid. Peptides were first loaded onto a nanotrap column (ThermoFisher PepMap C18, 5 µm, 100Å, 20 mm x 100 µm I.D.), then eluted onto a reversed phase Easy-Spray column (ThermoFisher PepMap C18, 3 µm, 100Å, 15 cm x 75 µm I.D.) using a linear 120-min gradient of acetonitrile (2-50%) containing 0.1% formic acid at 300 nL/min flowrate. The eluted peptides were sprayed into the Fusion Orbitrap with spray voltage and ion transfer tube temperature set at 1.8 kV and 250ºC, respectively. For identifications of N-linked glycosylation, DDA-MS2 was HCD (step collision energy of 20, 30, 40) followed with targeted mass trigger (204.0867 & 366.1396 within ± 15 ppm mass tolerance and among top 20 abundant ions) to subsequent EThcD (with ETD supplemental activation with SA collision energy 25). Automatic gain control (AGC) targets and maximum injection times were set as “standard” and “auto”, respectively.

Tandem mass spectra were searched with proteome discoverer 2.4 using the AChE-Fc fusion protein sequences and the Uniprot Cricetulus griseus (Chinese hamster) proteome for reference. Occupied N-glycan sites were identified by searching the tandem mass spectra in Protein Metrics Byologic using the protein sequences of AChE-Fc, and AChE(W86A)-Fc, and a glycan database of N-glycan 182 human no multiple fucose which contained all major species of N-glycans identified by permethylated N-glycan analysis. Peak areas of all glycopeptides were obtained from extracted ion chromatograms (XICs) and converted to % total glycopeptide to determine glycan site microheterogeneity. Occupancy of each glycan site was calculated by dividing the peak area of glycopeptide by the total sum of native peptides and glycopeptides at each site.

*Thermal denaturation and aggregation*

To determine the non-equilibrium melting temperature (T_m_) and aggregation temperature (T_agg_), purified AChE fusion proteins (1 mg/mL) were subjected to thermal ramping between 20°C and 95°C at 1°C increments while monitoring the intrinsic fluorescence using differential scanning fluorimetry (UNcle, Unchained Labs) in accordance with the manufacturer’s protocol and Compass software version 2. The T_m_, onset of Tm (T_m_ onset), and T_agg_ at 266 nm were calculated using the Compass software version 2.

*Circular Dichroism*

Far ultraviolet circular dichroism was performed using a JASCO-1700 equipped with a EXOS liquid cooling system and temperature-controlled six-cell holder. The 0.4 mg/mL protein samples were buffer exchanged using the 30 kDa molecular weight cutoff filters into 0.1M sodium phosphate pH 7.4 buffer, and wavelength scans were measured using a 0.5 mm path-length quartz cuvettes. Data acquisition was performed using range of 260–185 nm at 20°C, 45°C, 65°C, and 80°C, temperature ramp between measurements being 6 °C/min, 0.2 nm data pitch, 20 mdeg/0.05 dOD CD scale, 1 nm bandwidth, 10 nm NIR bandwidth, 50 nm/min continuous scanning for 4 accumulations. Each protein batch was run in triplicate at 20°C and melt from 20 to 80°C was performed once on each batch and results were averaged into single data set.

The spectral ellipticity data was transformed into mean residual ellipticity (Δε_MR_) data assuming dimer and molar concentration of 0.00000232, 1570 amino acids in unit, and 0.05 cm pathlength using the following equations where θ= machine units, ΔA= absorbance, and Δε_MR_= mean residual ellipticity.

$$\theta= \frac{mdeg}{1000}$$

$$\Delta A=\frac{\theta}{32.982}$$

$${\Delta\varepsilon}_{MR}= \frac{\Delta A}{Concentration \left( M \right)*number of amino acid residues*pathlength (cm)}$$

The ΔεMR data was used in BeStSel with scaling factor of 1 to estimate secondary structure components in the samples [2].

*AChE activity*

The commercial colorimetric assay (Cat # MAK119-KT, Millipore Sigma) was modified to determine the AChE activity of the fusion proteins. Briefly, the Calibrator (200 U/L) was serial diluted from 200 U/L to 100 U/L, 50 U/L, 25 U/L, and 12.5 U/L using assay buffer to establish a 5-point linear regression analysis that was used to establish the AChE activity of the diluted fusion proteins by mass (mg/L).

*AChE inhibition assay*

Briefly, AChE activity was determined using a BioTek plate (96 well) reader using a modification of the Ellman et al. method. The diluted fusion protein was suspended in 158 µl 50mM Tris buffer (pH 7.4) for each well at the predetermined concentration (5 µg protein/ml well) and warmed to 37°C. A vehicle to deliver the organophosphate, paraoxon, for the subsequent inhibition studies was determined by testing several combinations of aqueous and organic solvents. A final vehicle consisting of 1:1 (vol:vol), water:ethanol was selected. Vehicle, 2 µl, was added and the reaction mixture was incubated for 5 min for equilibration at 37°C. To correct for non-enzymatic hydrolysis of the substrate, 60 µM eserine sulfate was incubated in additional wells (blanks). The assay was initiated by the addition of 40 µl of a prewarmed mixture of 5 mM acetylthiocholine (ATCh, the substrate) in ethanol and 25 mM 5,5’-dithio bis(nitrobenzoic acid) (DTNB, the chromogen) dissolved in Tris buffer to yield final concentrations of 1 mM ATCh and 5 mM DTNB. The absorbance was recorded at 50 sec intervals for 8 minutes (for 10 readings) at 412 nm. Linearity of the reaction was determined to guarantee that substrate depletion did not occur.

*Paraoxon inhibition assay*

The above assay was conducted with the exception that paraoxon dissolved in vehicle (1:1 water/ethanol, vol:vol) or vehicle only was added and incubated for 5 min at various concentrations to obtain an AChE inhibition range of about 10-90%.

Fusion protein protection assay: To determine if the inactivated fusion protein, AChE(W86A)-Fc, could bind the OP (paraoxon) and serve as a bio-scavenger, an indirect AChE assay was conducted. Diluted AChE(W86A)-Fc fusion protein (5 µg protein/ml or 50 µg protein/ml) was incubated (5-20 min) at 37°C with the same concentrations of paraoxon used in the IC50 experiments above. Active diluted fusion protein (5 µg protein/ml) was added and incubated for 5 min as described above and AChE inhibition determined. A reduction in AChE inhibition compared to the active fusion protein alone would indicate binding by the inactive fusion protein.

**Supplementary Tables**

**Supplementary Table 1: CHO Cell line characterization during upstream production of AChE-Fc and AChE(W86A)-Fc.**

|  | CHO^AChE-Fc^ (n=4) | CHO^AChE(W86A)-Fc^  (n=5) |  |
| --- | --- | --- | --- |
| Viable cell density (10^6^/mL) | 17.8 | 12.61 | Ave. |
|  | 3.1 | 2 | S.D. |
|  | 17.2 | 15.8 | CV (%) |
| Viability on harvest day (%) | 94.3 | 94.6 | Ave. |
|  | 1 | 5.7 | S.D. |
|  | 1.29 | 6.1 | CV (%) |
| Protein titer of harvest media (mg/L) | 4.23 | 3.53 | Ave. |
|  | 0.31 | 0.52 | S.D. |
|  | 7.44 | 14.88 | CV (%) |

Ave. = Average, S.D. = Standard deviation, CV = Coefficient of Variation

**Supplementary Table 2: Efficiency of two-step downstream process for AChE-Fc and AChE(W86A)-Fc fusion protein purification.**

| Downstream Processing Efficiency | | | | | | | |
| --- | --- | --- | --- | --- | --- | --- | --- |
|  |  | AChE-Fc (n=4) | | | AChE(W86A)-Fc (n=5) | | |
|  | Calculations | Ave. | S.D. | CV (%) | Ave. | S.D. | CV (%) |
| AFP in harvest media (HM) per liter | N/A | 4.2 mg | 0.1 | 3.3 | 3.7 mg | 0.4 | 10.2 |
| Protein A elude (PAE) | N/A | 2.4 mg | 0.1 | 4.0 | 2.3 mg | 0.8 | 34.6 |
| Protein A recovery (PAR) | PAE/HM | 56 % | 4 % | 6.9 | 62 % | 12 % | 19.3 |
| AChE IEX elude (IEX) | N/A | 1.5 mg | 0.3 | 21.5 | 1.4 mg | 0.6 | 43.5 |
| IEX recovery (IER) | IEX/PAE | 64 % | 13% | 22.2 | 58 % | 14 % | 24.1 |
| Downstream recovery | PAR *IER | 36 % | 6 % | 19.9 | 37 % | 13 % | 35.1 |

Ave. = Average, S.D. = Standard deviation, CV = Coefficient of Variation

**Supplementary Table 3: Relative percentages of N-linked glycans identified using AChE-Fc from at least 3 batches performed in triplicate.**

| Glycan Name | Glycan Mass | Glycan Structure | Ave. | S.D. | CV |
| --- | --- | --- | --- | --- | --- |
| M5 | 1595.814 | 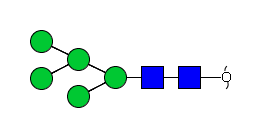 | 0.29% | 0.0017 | 58.3433 |
| FA2 | 1851.956 | 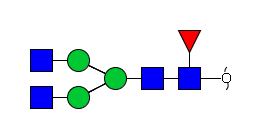 | 36.41% | 0.0529 | 14.5171 |
| FA2G1 | 2056.056 | 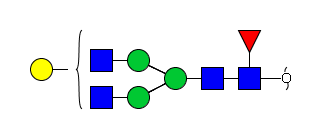 | 25.80% | 0.0326 | 12.6258 |
| FA3 | 2097.083 | 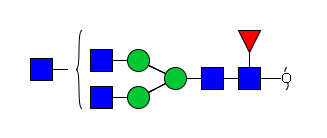 | 0.60% | 0.0011 | 18.5654 |
| FA2G2 | 2260.156 | 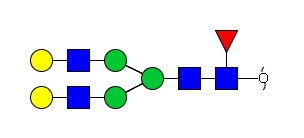 | 3.12% | 0.0026 | 8.1938 |
| FA3G1 | 2301.182 | 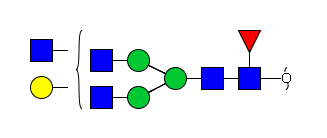 | 0.52% | 0.0018 | 34.7734 |
| FA4 | 2342.209 | 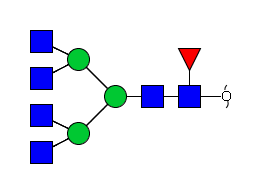 | 0.06% | 0.0009 | 150.0392 |
| FA2G1S1 | 2417.230 | 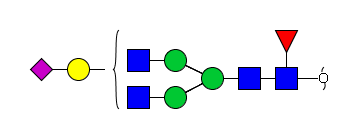 | 1.96% | 0.0043 | 21.8192 |
| FA2G2S1 | 2621.329 | 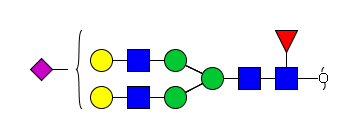 | 7.53% | 0.0128 | 16.9652 |
| FA3G1S1 | 2662.356 | 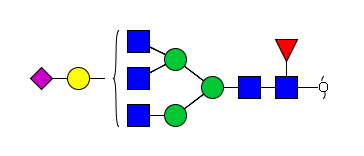 | 0.19% | 0.0013 | 66.1138 |
| FA2G2S2 | 2982.503 | 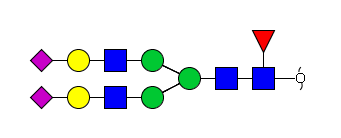 | 15.97% | 0.0428 | 26.8068 |
| FA3G3S1 | 3070.556 | 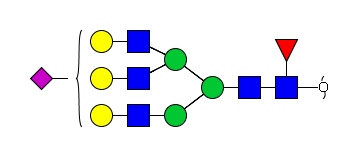 | 0.74% | 0.0016 | 21.5879 |
| FA3G2S2 | 3227.629 | 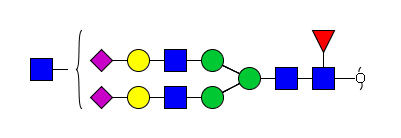 | 0.29% | 0.0009 | 30.1738 |
| FA3G3S2 | 3431.729 | 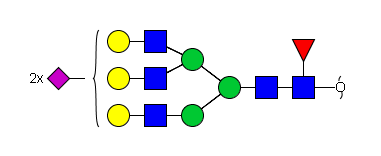 | 1.55% | 0.0059 | 37.6680 |
| FA4G2S2 | 3472.756 | 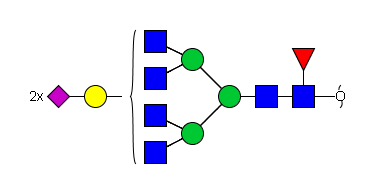 | 0.09% | 0.0009 | 97.6365 |
| FA4G3S2 | 3676.856 | 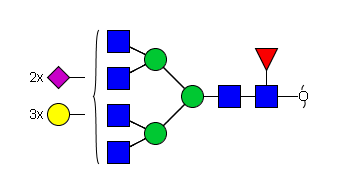 | 0.11% | 0.0008 | 76.9557 |
| FA3G3S3 | 3792.903 | 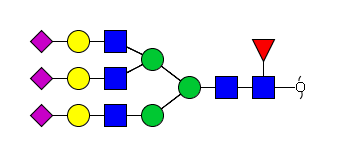 | 3.13% | 0.0130 | 41.5007 |
| FA4G4S2 | 3880.955 | \| 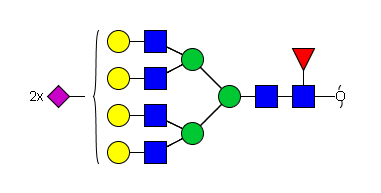 \| \| --- \| | 0.44% | 0.0016 | 35.5425 |
| FA4G3S3 | 4038.001 | 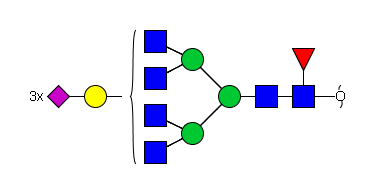 | 0.09% | 0.0002 | 22.2249 |
| FA4G4S3 | 4242.101 | 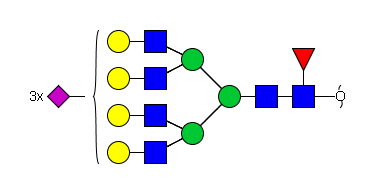 | 0.51% | 0.0019 | 37.3211 |
| FA4G4S4 | 4603.274 | \| 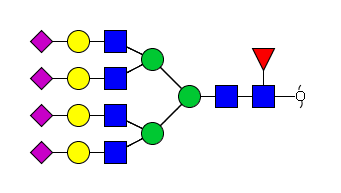 \| \| --- \| | 0.58% | 0.0020 | 34.4564 |

S.D. = Standard Deviation, Green circles = mannose, Yellow circles = Gal, Blue Square = GlcNAc, Red Triangle = fucose, Pink diamond = Neu5Ac, Ave. = Average (n=9), S.D. = Standard deviation, CV = Coefficient of Variation

**Supplementary Table 4: Relative percentages of N-linked glycans identified using AChE(W86A)-Fc from at least 3 batches performed in triplicate.**

| Glycan Name | Glycan Mass | Glycan Structure | Ave. | S.D. | CV |
| --- | --- | --- | --- | --- | --- |
| M5 | 1595.814 | 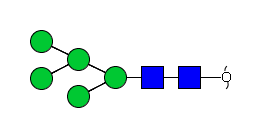 | 0.57% | 0.0008 | 14.7343 |
| FA2 | 1851.956 | 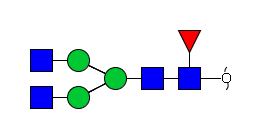 | 43.61% | 0.0395 | 9.0551 |
| FA2G1 | 2056.056 | 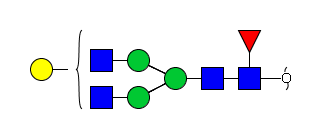 | 22.03% | 0.0147 | 6.6582 |
| FA3 | 2097.083 | 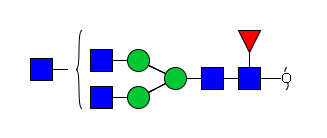 | 0.68% | 0.0018 | 27.1673 |
| FA2G2 | 2260.156 | 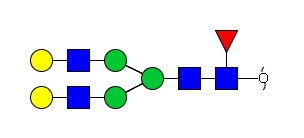 | 1.37% | 0.0053 | 38.8217 |
| FA3G1 | 2301.182 | 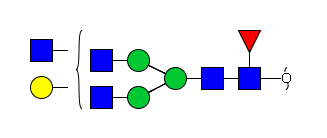 | 0.21% | 0.0012 | 57.1973 |
| FA4 | 2342.209 | 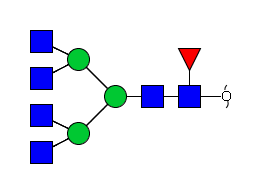 | 0.19% | 0.0021 | 113.8894 |
| FA2G1S1 | 2417.230 | 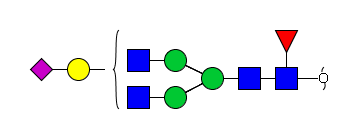 | 1.93% | 0.0036 | 18.7543 |
| FA2G2S1 | 2621.329 | 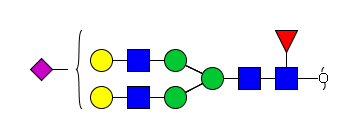 | 3.01% | 0.0049 | 16.2718 |
| FA3G1S1 | 2662.356 | 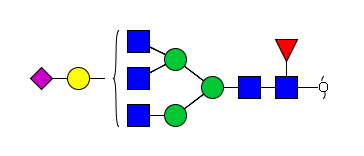 | 0.11% | 0.0016 | 150.8638 |
| FA2G2S2 | 2982.503 | 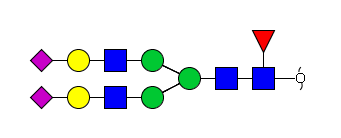 | 17.50% | 0.0229 | 13.0954 |
| FA3G3S1 | 3070.556 | 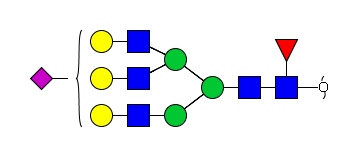 | 0.01% | 0.0003 | 300.0000 |
| FA3G2S2 | 3227.629 | 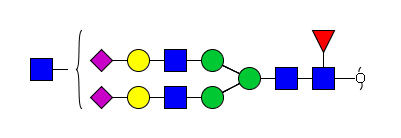 | 0.17% | 0.0015 | 86.4246 |
| FA3G3S2 | 3431.729 | 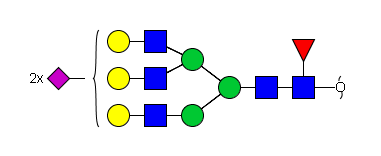 | 0.41% | 0.0014 | 34.7135 |
| FA4G2S2 | 3472.756 | 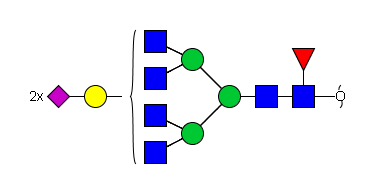 | 0.31% | 0.0021 | 68.6635 |
| FA4G3S2 | 3676.856 | 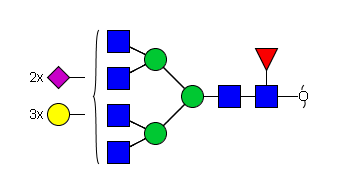 | 0.01% | 0.0002 | 300.0000 |
| FA3G3S3 | 3792.903 | 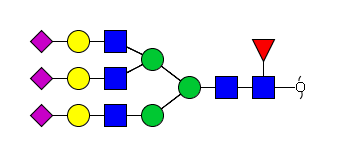 | 3.74% | 0.0076 | 20.2074 |
| FA4G4S2 | 3880.955 | 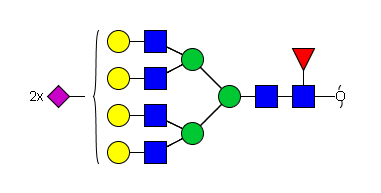 | 0.04% | 0.0005 | 139.6289 |
| FA4G3S3 | 4038.001 | 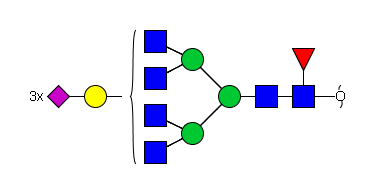 | 0.27% | 0.0012 | 44.0532 |
| FA4G4S3 | 4242.101 | 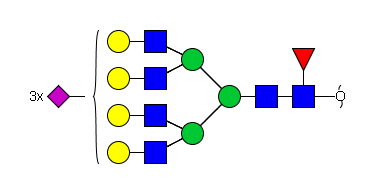 | 0.440% | 0.0015 | 34.8112 |
| FA4G4S4 | 4603.274 | 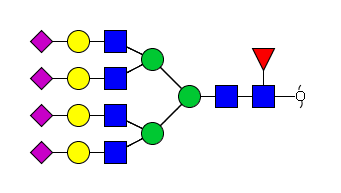 | 2.97% | 0.0063 | 21.2305 |

S.D. = Standard Deviation, Green circles = mannose, Yellow circles = Gal, Blue Square = GlcNAc, Red Triangle = fucose, Pink diamond = Neu5Ac, Ave. = Average (n=9), S.D. = Standard deviation, CV = Coefficient of Variation

**Supplementary Table 5: Results, degrees of freedom, factors, and figures for statistical analyses.**

| **Student's T Test** | | | | |
| --- | --- | --- | --- | --- |
| Test | Figure | F,DFn Dfd | *p* value | |
| AChE activity between AChE-Fc and AChE(W86A)-Fc | Figure 5a | 13494, 8, 8 | < 0.0001 | |
| AChE activity of AChE-Fc before and after thermal stress | Figure 5b | 34.38, 8, 8 | < 0.0001 | |
|  |  |  |  |  |
| **Two-Way ANOVA** | | | | |
| Test | Figure | Factors | F (DFn, DFd) | *p* value |
| N-Glycan profile differences between AChE-Fc and AChE(W86A)-Fc | Figure 2b | Interaction | F (20, 336) = 11.01 | < 0.0001 |
|  |  | N-glycan | F (20, 336) = 894.2 | < 0.0001 |
|  |  | Protein | F (1, 336) = 0.02007 | 0.8874 |
| N370 site macro-heterogeneity differences between AChE-Fc and AChE(W86A)-Fc | Figure 2e | Interaction | F (36, 148) = 31.41 | P<0.0001 |
|  |  | N-glycan | F (36, 148) = 61.06 | P<0.0001 |
|  |  | Protein | F (1, 148) = 0.0006090 | P=0.9803 |
| N484 site macro-heterogeneity differences between AChE-Fc and AChE(W86A)-Fc | Figure 2f | Interaction | F (34, 140) = 6.375 | P<0.0001 |
|  |  | N-glycan | F (34, 140) = 160.0 | P<0.0001 |
|  |  | Protein | F (1, 140) = 0.0001973 | P=0.9888 |
| N654 site macro-heterogeneity differences between AChE-Fc and AChE(W86A)-Fc | Figure 2g | Interaction | F (15, 64) = 41.38 | P<0.0001 |
|  |  | N-glycan | F (15, 64) = 2123 | P<0.0001 |
|  |  | Condition | F (1, 64) = 3.567e-005 | P=0.9953 |
| AChE-Fc - Secondary structure differences between temperatures | Figure 3e | Interaction | F (8, 30) = 2.121 | P=0.0651 |
|  |  | Secondary Structure | F (4, 30) = 416.9 | P<0.0001 |
|  |  | Temperature | F (2, 30) = 2.134e-006 | P>0.9999 |
| AChE(W86A)-Fc - Secondary structure differences between temperatures | Figure 3f | Interaction | F (8, 15) = 8.044 | P=0.0003 |
|  |  | Secondary Structure | F (4, 15) = 780.4 | P<0.0001 |
|  |  | Temperature | F (2, 15) = 0.5850 | P=0.5693 |
| AChE-Fc thermal stress induced fragmentation  (Reduced) | Figure 4c (Reduced) | Interaction | F (6, 72) = 303.9 | < 0.0001 |
|  |  | Fragments and peaks | F (2, 72) = 313.9 | < 0.0001 |
|  |  | Condition | F (3, 72) = 0.2014 | 0.8951 |
| AChE-Fc thermal stress induced fragmentation  (Non-reduced) | Figure 4c (Non-reduced) | Interaction | F (3, 46) = 276.7 | P<0.0001 |
|  |  | Fragments and peaks | F (1, 46) = 134.5 | P<0.0001 |
|  |  | Condition | F (3, 46) = 0.01423 | P=0.9976 |
| AChE(W86A)-Fc thermal stress induced fragmentation  (Reduced) | Figure 4d (Reduced) | Interaction | F (6, 60) = 69.86 | < 0.0001 |
|  |  | Fragments and peaks | F (2, 60) = 50.44 | < 0.0001 |
|  |  | Condition | F (3, 60) = 0.01067 | 0.9985 |
| AChE(W86A)-Fc thermal stress induced fragmentation  (Non-reduced) | Figure 4d (Non-reduced) | Interaction | F (6, 60) = 122.8 | P<0.0001 |
|  |  | Fragments and peaks | F (2, 60) = 0.2879 | P=0.7509 |
|  |  | Condition | F (3, 60) = 0.004193 | P=0.9996 |

**Supplementary Figures**

**
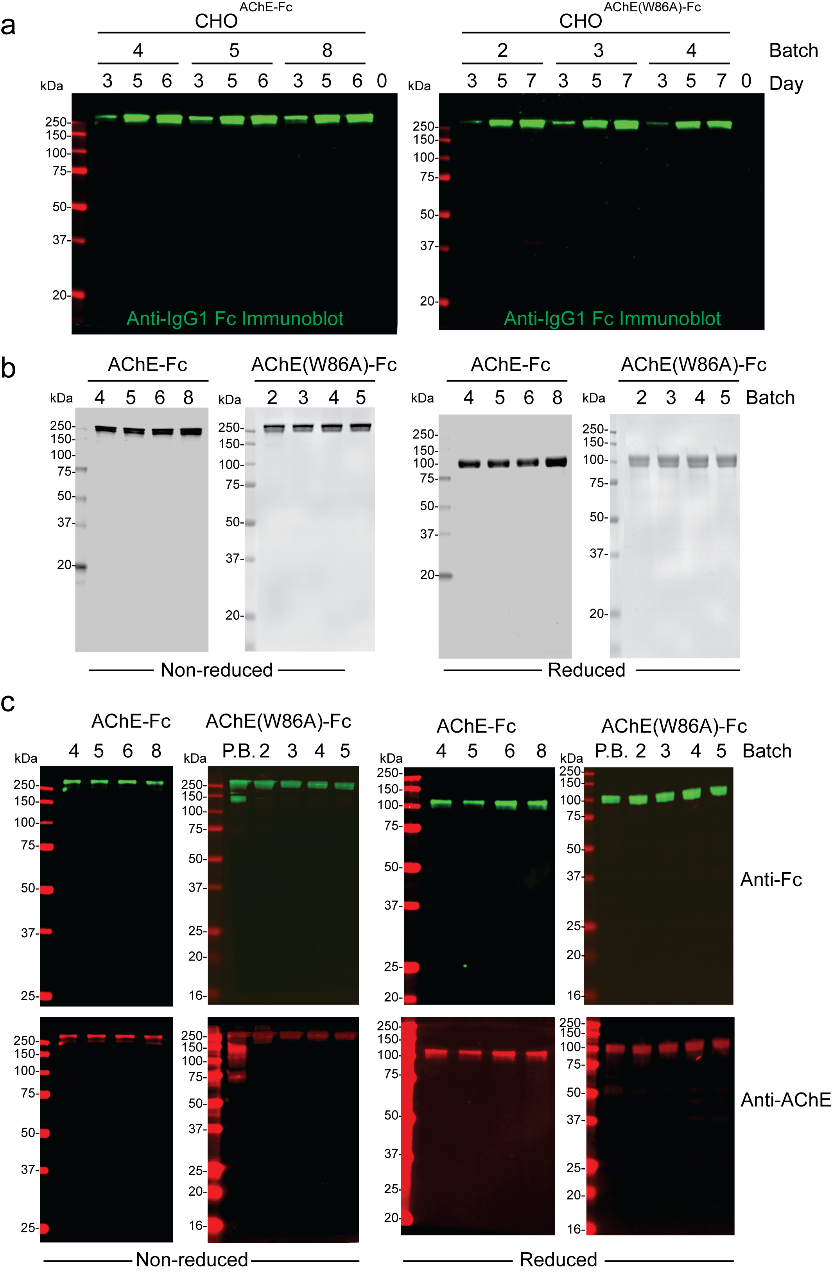
**

**Supplementary Figure 1: Purity and identity characterization of the secreted and purified AChE fusion proteins.** (a) Representative IgG-Fc immunoblot of harvest media prior to downstream processing on day 3, 5, and 7 from three different batches of AChE-Fc and AChE(W86A)-Fc. b) Coomassie-stained SDS-PAGE gels of purified AChE fusion proteins) under non-reducing and reducing conditions. a) IgG-Fc and AChE immunoblots using purified AChE fusion proteins (under non-reducing and reducing conditions. Preliminary Batch (P.B.) was not selected for further physicochemical and biological studies.

**
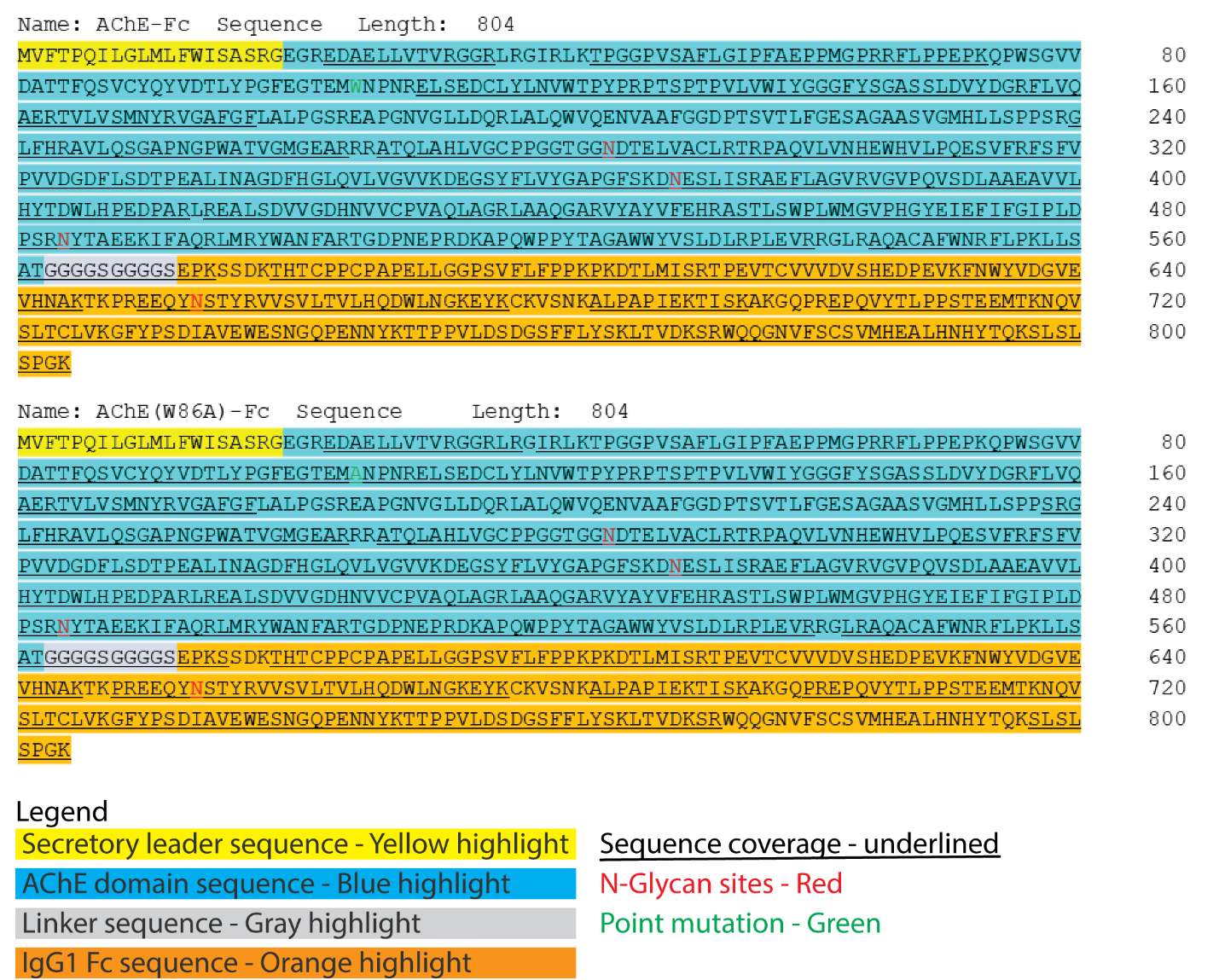
**

**Supplementary Figure 2: Peptide map coverage of the AChE fusion proteins with N-glycan sites, point mutation site, and sequence domains.**

**
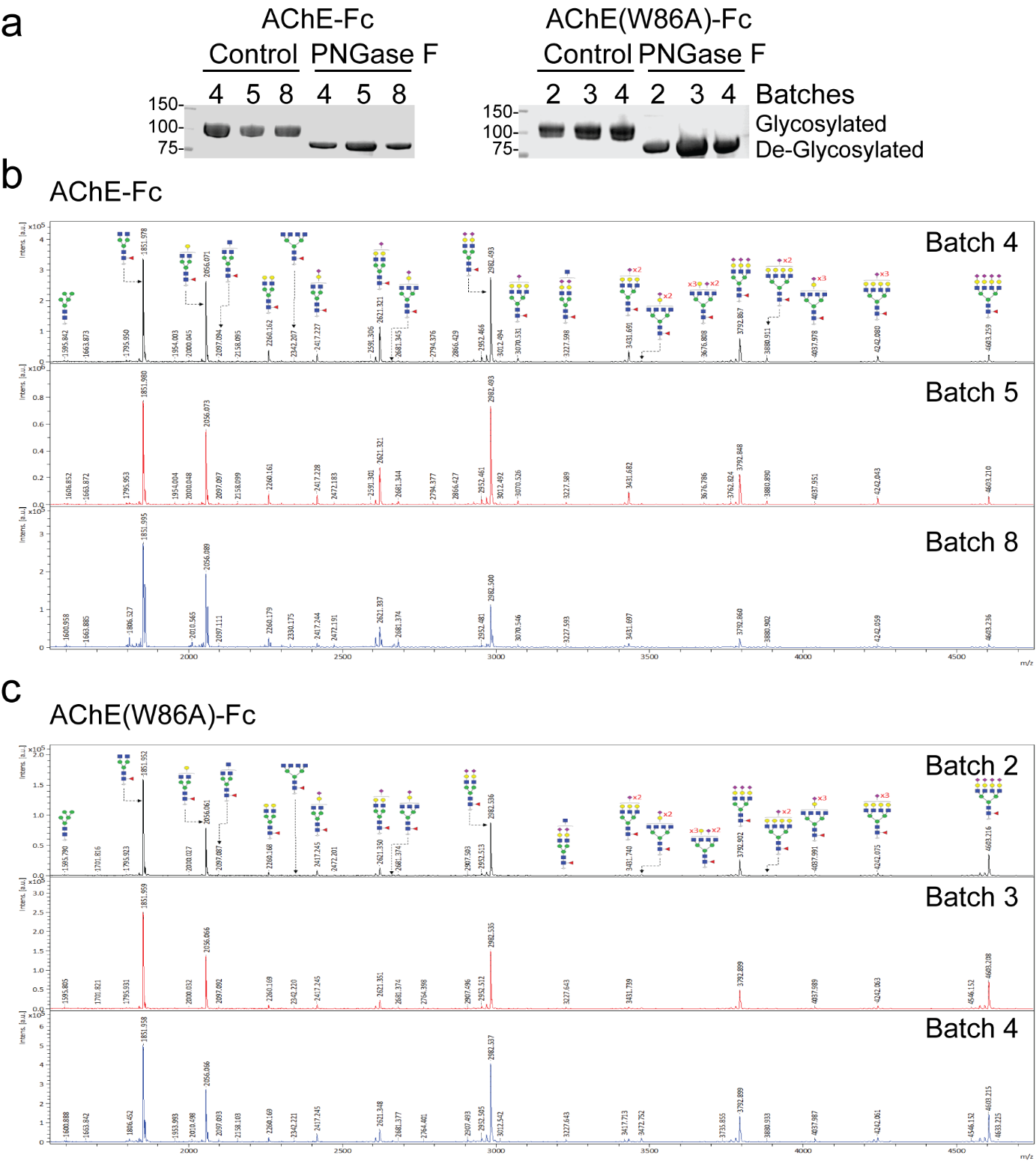
****Supplementary Figure 3: N-glycan profiles of AChE-Fc and AChE(W86A)-Fc.** (a) Coomassie-stained SDS-PAGE gel of AChE-Fc and AChE(W86A)-Fc with and without PNGase F treatment to release N-glycans under reducing conditions. (b &c) Representative N-glycan spectra of AChE-Fc (b) and AChE(W86A)-Fc (c) from three different batches.


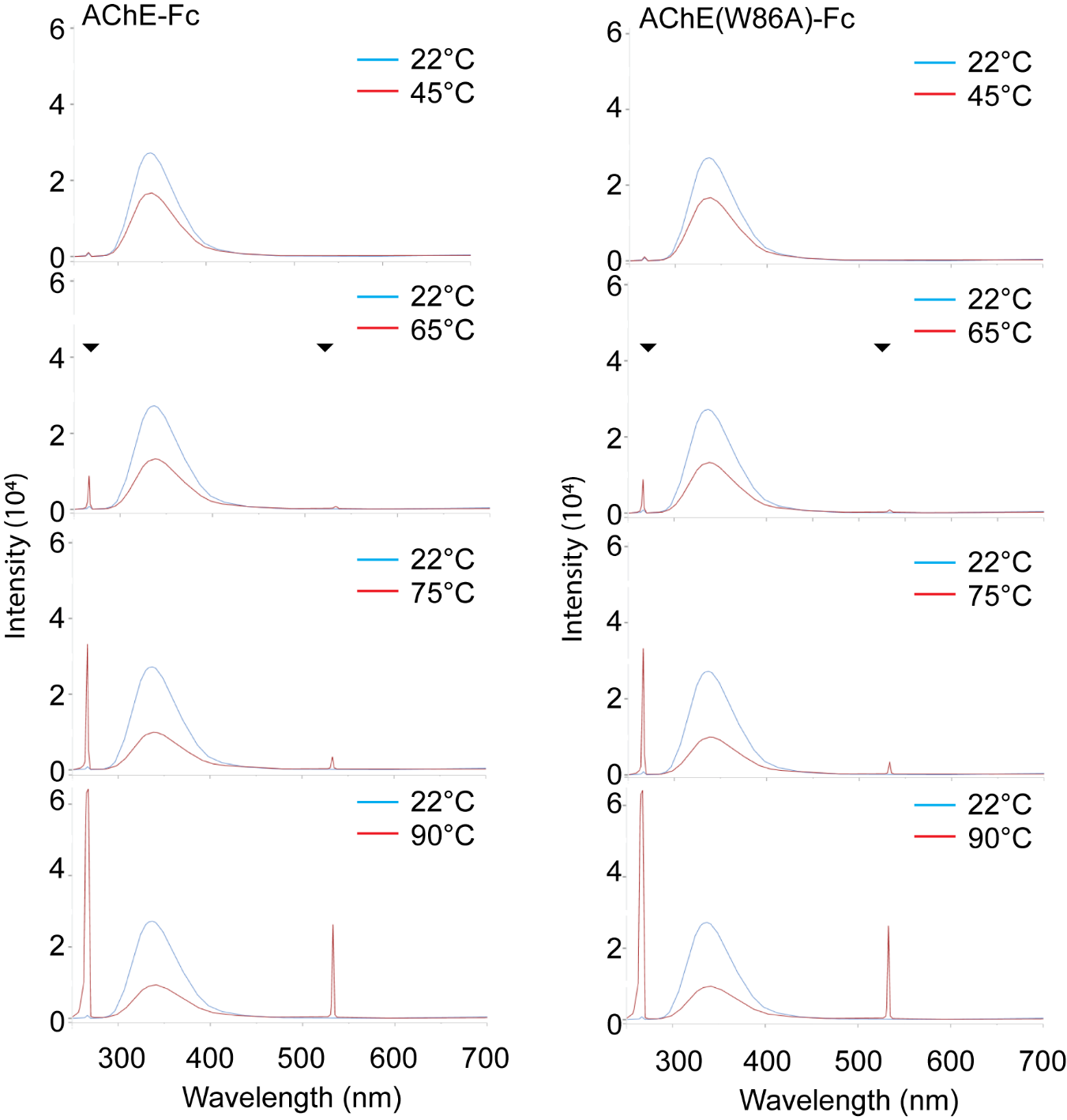


**Supplementary Figure 4: Static intrinsic fluorescent changes of AChE- Fc and AChE(W86A)-Fc at 22, 65, 75, and 90°C.** Spectral changes of the AChE-Fc fusion proteins during thermal ramping.


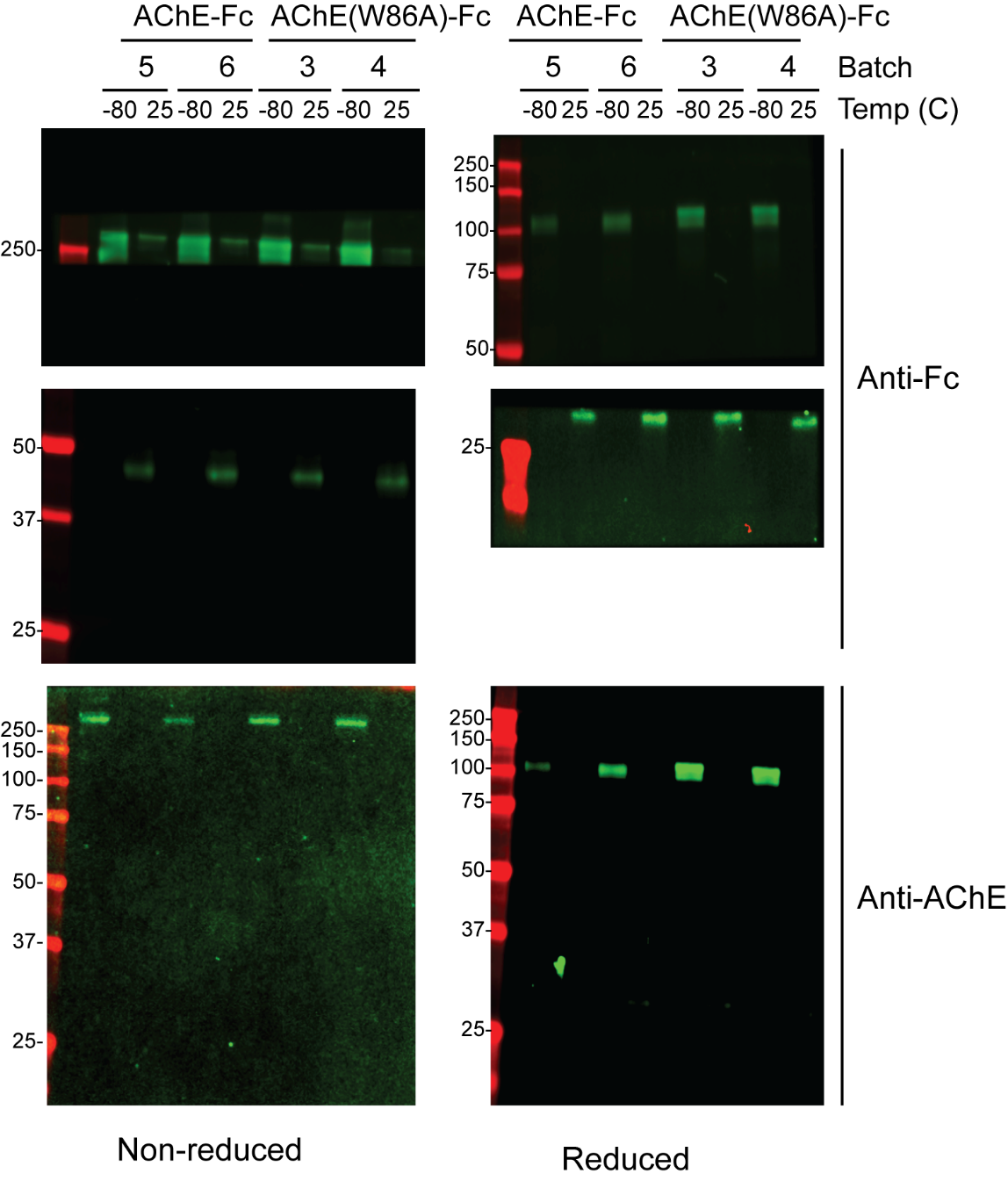


**Supplementary Figure 5: Raw immunoblots of AChE fusion proteins subjected to storage for 7 days at 25°C.** The anti-IgG Fc immunoblot membrane was cut below 150 and above 50 kDa prior to imaging to improve detection of IgG1 Fc bands. The AChE immunoblot membrane remained intact for imaging.

**References**

1. Wisniewski, J.R., *Filter-Aided Sample Preparation for Proteome Analysis.* Methods Mol Biol, 2018. **1841**: p. 3-10.

2. Micsonai, A., et al., *BeStSel: a web server for accurate protein secondary structure prediction and fold recognition from the circular dichroism spectra.* Nucleic Acids Res, 2018. **46**(W1): p. W315-W322.
